# An extraction-free, lyophilized one-pot RAA-CRISPR assay for point-of-care testing of *Haemophilus influenzae*

**DOI:** 10.1128/jcm.00535-25

**Published:** 2025-09-23

**Authors:** Yaling Cao, Junwen Wang, Zihao Fan, Ling Xu, Zhenzhen Pan, Yinkang Mo, Qiwen Yang, Jing Huang, Feng Ren

**Affiliations:** 1 Beijing Institute of Hepatology/Beijing Youan Hospital, Capital Medical University 12517, Beijing, China; 2 Department of Clinical Laboratory, Chui Yang Liu Hospital 512709, Beijing, China; 3 Department of Clinical Laboratory, State Key Laboratory of Complex Severe and Rare Diseases, Peking Union Medical College Hospital, Chinese Academy of Medical Sciences 670119, Beijing, China; 4 Key Laboratory of Pathogen Infection Prevention and Control, Peking Union Medical College, Ministry of Education 12543 , Beijing, China; 5 Department of Infection Control, Peking Union Medical College Hospital, Chinese Academy of Medical Sciences & Peking Union Medical College 12501, Beijing, China; Endeavor Health, Evanston, Illinois, USA

**Keywords:** *Haemophilus influenzae*, CRISPR/Cas13a, one-pot assay, point‐of‐care testing (POCT), molecular diagnosis

## Abstract

Timely and accurate diagnosis is essential for the effective treatment of *Haemophilus influenzae* (HI) infection. Notably, a rapid point‐of-care testing (POCT) method for the diagnosis of HI DNA is still lacking. The aim of this study was to establish a one-pot detection device for HI DNA detection using CRISPR/Cas13a that would be applicable for POCT. We established a two-step polymerase chain reaction (PCR)-CRISPR assay, a two-step recombinase-aided amplification (RAA)-CRISPR assay, and a one-pot RAA-CRISPR assay. Additionally, reagent lyophilization, rapid lysis technology, and a one-pot detection device were integrated to construct an extraction-free one-pot detection device, named EFORCA (extraction-free one-pot RAA-CRISPR/Cas13a assay). Validation was performed on 90 simulated samples and 77 clinical samples. The two-step PCR/RAA-CRISPR assay for HI DNA detection was established and optimized, with a detection limit of 1 copy/μL and 100% specificity. The sensitivity and specificity of the two-step PCR-CRISPR assay for the 90 simulated clinical samples were 96.7% and 95%, respectively, and for the 77 clinical samples, 97.5% and 100%, respectively. The one-pot RAA-CRISPR assay and the EFORCA detected 50 and 25 CFU/mL HI in bacterial suspensions within 30 min, respectively. The valuation results for 77 clinical samples demonstrated that the sensitivity of both methods was 95%, which was greater than the sensitivity of quantitative real-time PCR (qPCR) (92.5%) with the same specificity of 100%. We developed an extraction-free one-pot RAA-CRISPR/Cas13a assay for HI detection, which effectively performs a POCT test and is a valuable tool for the early detection and monitoring of HI infection.

**IMPORTANCE:**

Timely and accurate diagnosis is essential for the effective treatment of *Haemophilus influenzae* (HI) infection. Notably, a rapid point‐of-care testing (POCT) method for the diagnosis of HI DNA is still lacking. In this study, sensitive two-step polymerase chain reaction (PCR)-CRISPR and recombinase-aided amplification (RAA)-CRISPR assays were developed, followed by the establishment of the one-pot RAA-CRISPR assay through integration of RAA, CRISPR, and rapid lysis for HI detection. Additionally, reagent lyophilization, rapid lysis technology, and a one-pot detection device were integrated to construct the extraction-free one-pot RAA-CRISPR/Cas13a assay (EFORCA). With its high sensitivity, specificity, rapid turnaround time, and operational simplicity, this assay shows great potential as a practical diagnostic tool for HI infection in small laboratory settings.

## INTRODUCTION

*Haemophilus influenzae* (HI) is a Gram-negative bacterium that is categorized into typeable (encapsulated) and nontypeable (nonencapsulated) strains (1–3). Nontypeable *Haemophilus influenzae* (NTHi) is responsible for 20% to 40% of childhood otitis media and respiratory tract infections, such as pneumonia and acute bronchitis (4). In severe cases, NTHi infection can lead to acute exacerbation of chronic obstructive pulmonary disease (5, 6). Type b *Haemophilus influenzae* (Hib) is the most virulent strain and can cause acute/invasive diseases, such as sepsis, pneumonia, meningitis, cellulitis, epiglottitis, and suppurative arthritis, which involve bloodstream infection and hematogenous dissemination (7–9). Invasive diseases caused by Hib infection occur mainly in infants and young children and were once the principal cause of bacterial meningitis in children (10). Nevertheless, studies have indicated that Hib can infect individuals of all age groups, with a higher probability among those with a compromised immune system (8).

With the introduction and widespread use of Hib vaccines, the prevalence of Hib and the incidence of related diseases have notably decreased (11, 12). However, for children infected with Hib, clinical manifestations are often severe, accompanied by a high mortality rate for those with intracranial infection and numerous sequelae, which still cannot be disregarded (13, 14). Hence, early diagnosis of HI infection and the administration of antibiotic treatment are vital.

At present, traditional bacterial culture processes impose high environmental demands, are time-consuming, and have a low success rate in isolating single colonies (15). Distinguishing HI from other Haemophilus species is challenging and thus fails to meet the requirements for rapid detection. The emergence of molecular detection techniques has offered some effective tools to address this issue. Nevertheless, certain deficiencies remain in the clinical application of these methods. Quantitative real-time PCR (qPCR) technology is widely utilized in clinical practice because of its high sensitivity and specificity (16). However, for samples with low target gene concentrations, missed detections may still occur, and detection relies on the establishment of standard curves. The 16S rRNA sequencing is a high-throughput sequencing technology targeting the bacterial small ribosomal subunit (16S rRNA gene), commonly used for microbial identification. However, it requires sophisticated instrumentation and professional technicians (17). Matrix-assisted laser desorption/ionization time-of-flight mass spectrometry (MALDI-TOF MS) enables rapid detection of the bacterial proteome, but in most instances, relatively pure colonies must be obtained through bacterial culture as a foundation. Both of these methods require expensive equipment and professional technicians working in clinical laboratories (18, 19).

In contrast, loop-mediated isothermal amplification (LAMP) typically reacts at 60°C–65°C, whereas recombinase-aided amplification (RAA) can be performed at 37°C. Both techniques eliminate the need for sophisticated instrumentation, making them particularly suitable for on-site diagnosis in community or resource-scarce medical institutions (16, 20). Compared with LAMP, RAA requires fewer primer pairs, thereby minimizing cross-reactions, and it has been employed in the detection of various pathogens (21). Nevertheless, RAA assays are inherently susceptible to aerosol contamination, which may compromise the reliability of the experimental results (22, 23).

The clustered regularly interspaced short palindromic repeats (CRISPR)/CRISPR-associated protein (Cas) system has been extensively employed in the establishment of nucleic acid detection platforms in recent years. In 2017, Science reported that the Feng Zhang laboratory at the Massachusetts Institute of Technology in the United States initially combined CRISPR/Cas13 detection technology with recombinase polymerase amplification technology to establish the nucleic acid diagnostic platform SHERLOCK (24). RAA-CRISPR/Cas technology has subsequently been applied for the detection of various pathogens, such as severe acute respiratory syndrome coronavirus 2 and dengue virus (23). This method is rapid, sensitive, and specific. However, there are certain challenges associated with the process of combining RAA with the CRISPR/Cas13 system. Specifically, aerosol contamination is prone to occur during RAA amplification because the reaction is performed at room temperature and because of the high amplification efficiency. Moreover, when attempting to integrate the two steps into a single-tube reaction, the desired sensitivity cannot be achieved because of interference between the RAA and CRISPR reagents (23). Therefore, novel, rapid, sensitive, and specific molecular detection methods for HI are urgently needed.

In this study, we designed a new two-step PCR/RAA CRISPR assay for the precise detection of HI DNA and further integrated RAA, CRISPR, and rapid lysis into a one-pot RAA-CRISPR assay. In the detection system, after PCR/RAA primers specifically amplify the target sequence, the Cas protein and crRNA recognize and bind to the target sequence, ensuring detection specificity while enhancing sensitivity. Finally, by introducing a rapid sample lysis step, a small one-pot device, and a lateral flow strip to develop the extraction-free one-pot RAA-CRISPR/Cas13a assay (EFORCA), this assay is anticipated to achieve point-of-care detection of HI and be widely applicable.

## MATERIALS AND METHODS

### Study design

**Fig 1:**
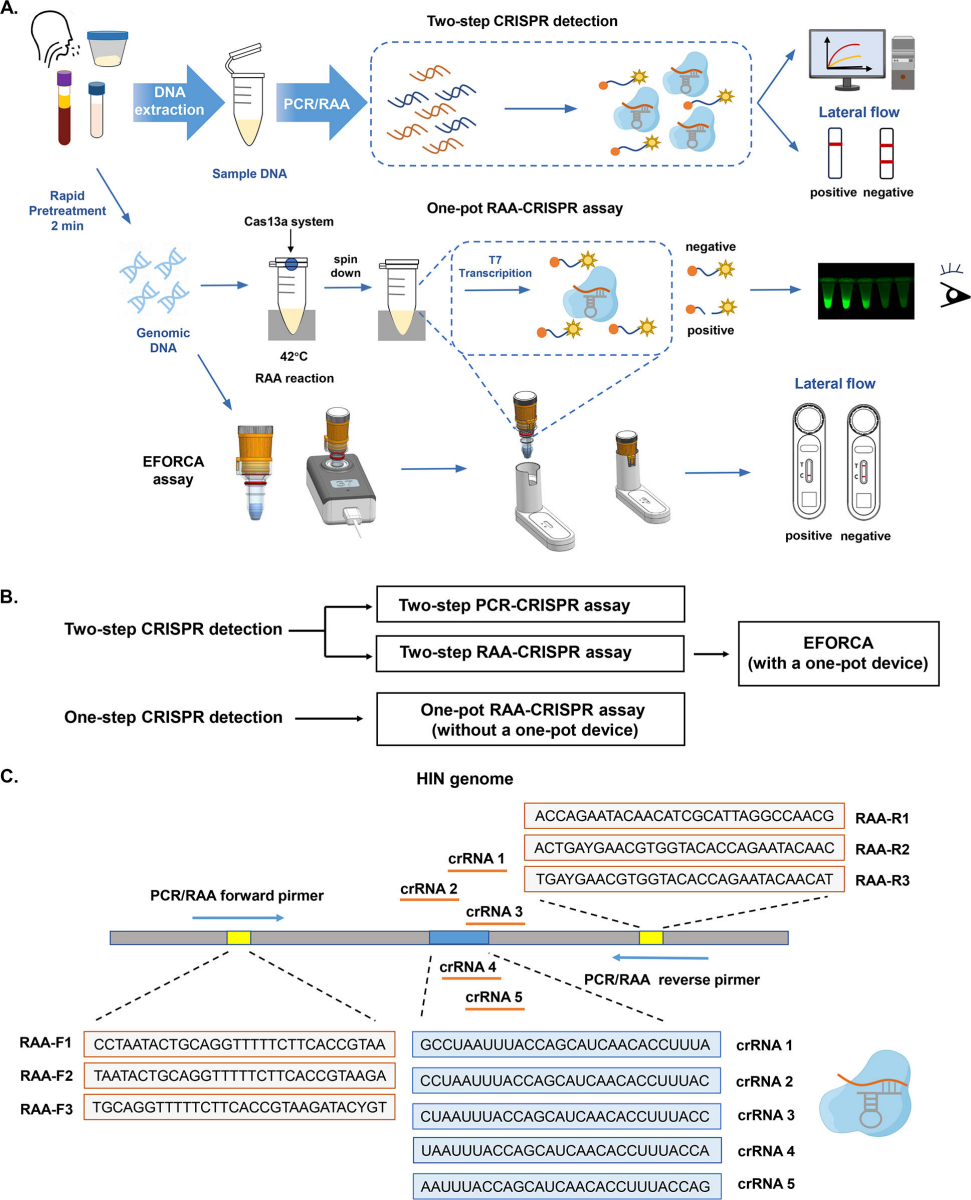
Schematic of CRISPR/Cas13a assays for HI detection. (**A**) Schematic of the workflow for HI detection (two-step PCR-CRISPR assay, two-step RAA-CRISPR assay, one-pot RAA-CRISPR assay, and EFORCA). (**B**) The summary of the methods established in this study. (**C**) Specific PCR/RAA primers and crRNA sequences were designed for the *ompP6* gene. The blue area represents the crRNA sequence, and the gray area represents the RAA primer sequence.

In this study, we designed and screened specific PCR/RAA primers and crRNAs that were constructed to accurately detect HI DNA via a two-step PCR-CRISPR assay and a two-step RAA-CRISPR assay. Based on the two-step RAA-CRISPR assay, a one-pot RAA-CRISPR assay, and an EFORCA were designed for rapid diagnosis of HI infection in a format that allows on-site detection in small laboratories (Fig. 1A and B).

### Bacterial culture and sample pretreatment methods

From January to November 2024, 40 HI-positive and 37 HI-negative sputum samples were collected at Beijing Chuiyangliu Hospital. These positive samples were confirmed by culture on chocolate agar plates at 37°C and identified via MALDI-TOF MS (used as the diagnostic gold standard for HI identification in this study). All these samples had a volume greater than 1 mL, and the qualification of sputum samples was determined by sputum smear microscopy (the qualification criteria were defined as >25 white blood cells and <10 epithelial cells per low-power field). The samples were transported using ice packs and promptly stored at −20°C.

### Preparation of bacterial suspensions

The HI reference strains were cultured on chocolate agar at 37°C to obtain isolated colonies. A colony was inoculated into brain heart infusion broth and cultured in a constant-temperature shaking incubator (Crystal Instruments, USA) at 37°C with agitation at 220 rpm until the OD_600_ reached 0.5–0.6. The bacterial cells were then collected by centrifugation at 13,000 × *g* for 10 min using a high-speed refrigerated centrifuge (Eppendorf, Germany), followed by two washes with sterile phosphate-buffered saline (PBS). Finally, the bacterial cells were resuspended in sterile PBS to achieve a final concentration of 1 × 10⁶ CFU/mL. The solutions were diluted to the desired concentration with sterile PBS, and 500 µL was processed for DNA extraction.

The specificity assessment employed 16 bacterial strains, as detailed in Table S2. Primary cultures were grown overnight at 37°C to obtain isolated colonies. Individual colonies were then transferred to liquid broth and cultured until an OD_600_ of 0.5–0.6 was achieved. Bacterial cells were collected via centrifugation (13,000 × *g*, 10 min), subjected to two PBS washes, and finally resuspended in PBS. From each suspension, 500 µL was processed for DNA extraction and subsequently used for specificity analysis.

### Preparation of simulated samples

Serially diluted HI bacterial suspensions were added to sputum or plasma to obtain simulated HI samples with concentrations ranging from 1 × 10^6^ CFU/mL to 1 × 10^1^ CFU/mL, and 500 µL of each simulated sample was used for DNA extraction and rapid sample lysis processing.

### DNA extraction

The bacterial DNA was extracted with a bacterial genome extraction kit (Tiangen Biochemical Technology, Beijing, China), and the simulated clinical samples and clinical samples were extracted with a universal genomic DNA extraction kit (Tiangen Biochemical Technology, Beijing, China). The bacterial DNA was stored at −80°C.

### Preparation of HI genomic DNA

A 500 µL aliquot of the prepared bacterial suspension (1 × 10⁶ CFU/mL) was used for genomic DNA (gDNA) extraction, yielding purified gDNA. The DNA concentration was accurately quantified via digital droplet PCR (ddPCR). A 10-fold serial dilution was subsequently performed using nuclease-free sterile water to obtain concentrations ranging from 10^−^¹ to 10⁴ copies per microliter. The detailed protocols for ddPCR are provided in the Supplementary materials.

### Design and screening of PCR/RAA primers and crRNA

The outer membrane protein P6 gene (*ompP6*) of HI (reference sequence GenBank accession: KC332053.1) was identified as a target for HI DNA detection. Owing to its high specificity, this gene has been widely employed in previous studies for HI detection (15). We downloaded 52 *ompP6* gene sequences from the NCBI database and performed sequence alignment using MEGA7 software to identify conserved regions. From this sequence, 600 bp was selected for DNA synthesis (Supplementary materials). pUC57 served as the template for constructing artificial HI synthetic DNA (Beijing Biomed Gene Technology Co., Ltd., Beijing, China). We designed five crRNA sequences and multiple pairs of PCR/RAA primers for screening (the selected sequence fragments are all those with a conservation rate of over 95%) (Fig. 1C). Probes and primers for qPCR were also designed (Table S1). The length of the PCR primers is 18–23 base pairs (bp), while the length of the RAA primers is 29–33 bp (the difference in primer length is due to RAA technology performing rapid nucleic acid amplification under isothermal conditions, which necessitates specific primer length requirements to ensure proper primer-recombinase interaction and maintain amplification specificity).

After synthesizing these sequences, we used a 10^3^ concentration of synthetic DNA for screening. The formula for calculating the concentration of synthetic DNA is copies/μL = [6.02 × 10^23^ × DNA concentration (ng/μL) × 10^−9^]/[genomic DNA length (nt) × 660]. Synthetic DNA amplification was performed using different PCR/RAA primer combinations, with the optimal primer set selected by analyzing agarose gel electrophoresis results of amplification products (by observing the grayscale of the bands). Subsequently, the selected optimal primer pair was used for amplification, and CRISPR detection was performed on the amplified fragments using each of the five crRNAs individually. The most effective crRNA was determined through fluorescence signal analysis.

### Rapid sample lysis optimization

#### Plasma samples

The sample lysis conditions for the simulated samples containing 10^3^ CFU/mL were optimized by two treatments: (i) adding the lysis solution (Beisheng Jingze Biotechnology Co. Ltd., Jiangsu, China) directly (standing at room temperature for 1 min) and (ii) adding the lysis solution, followed by heating at 95°C for 1 min.

#### Sputum samples

The sputum samples were (i) added to the lysis solution directly (shaking for 1 min), (ii) treated with 4% NaOH (shaking for 1 min), followed by the addition of lysis solution (1 min). The DNA obtained by the above different processing methods was detected by RAA-CRISPR, and the appropriate cleavage method was selected according to the fluorescence signal value.

### One-pot RAA-Cas13a assay

The RAA amplification and CRISPR reactions were performed in a single tube, with the RAA reagents and partial CRISPR components pre-loaded at the bottom of the tube, while the key CRISPR elements (Cas13a protein and crRNA) were stored separately in the tube cap. After RAA amplification, a brief centrifugation mixes these components into the reaction mixture for CRISPR detection. The one-pot RAA-CRISPR assay employs fluorescence readout.

The total system volume was 30 µL and included the RAA amplification and Cas13a detection components. Nine microliters of RAA mixture was placed at the bottom of each tube, comprising 480 nM forward and reverse primers, buffer A, buffer B, NTP mix (10 mM, New England Biolabs), HEPES buffer (10 mM, Thermo Fisher Scientific), MgCl_2_ (10 mM), 250 nM quenched fluorescent RNA reporter solution (2,000 nM, RNase Alert, Thermo Fisher Scientific), and recombinant RNase inhibitor (40,000 U/mL, New England Biolabs). T7 RNA polymerase (50,000 U/mL, New England Biolabs) and 200 nM Cas13a/crRNA binary complex were added to the tube cap. A binary complex was formed by mixing Cas13a and crRNA at a 1.5:1 molar ratio (300 nM Cas13a + 200 nM crRNA). After 1 µL of the DNA template was added, the tube was gently covered, and the components at the bottom of the tube were mixed slowly and carefully. After being placed in a 37°C metal bath for 15 min, the reaction mixture was centrifuged by vortex centrifugation. The fluorescence signals were detected via a LightCycler 480 System (Hoffmann‒La Roche) every 2 min for 30 cycles at 37°C.

### Extraction-free one-pot RAA-CRISPR/Cas13a assay

The one-pot device was divided into the two-step RAA-CRISPR assay. The RAA and CRISPR reaction systems were prepared as freeze-dried balls and placed into different chambers of the device. The lyophilization reagents, mini-incubator, and one-pot assay device were produced and customized by MicroDiag Biomedicine Co., Ltd. (Jiangsu, China). The method used can be summarized as follows: first, samples were processed by adding lysis solution (sample to lysis solution 1:6), and the mixture was allowed to stand for 1 min to facilitate sample lysis and nucleic acid release. Fifty microliters of the lysed sample was added to tube 3 with a dosing pipette and mixed thoroughly, ensuring that the liquid was at the bottom of tube 3. Subsequently, 320 µL of diluent (nuclease-free sterile water) was carefully added to the CRISPR reaction chamber (tube 4) using a dosing pipette. The lid was then closed. A simple thermostat was then used to maintain a constant temperature (42°C) for 15 min. After amplification, the ring between tube 3 and tube 4 was pulled, placed vertically, and pressed from the top until all the liquid in tube 4 entered tube 3, ensuring that the liquid was at the bottom of the tube after thorough mixing. The reaction was carried out at 37°C for 10 min. Finally, the lateral flow strip was inserted into the detection unit to read the results (Fig. 5A). The detailed instructions for use are provided in the Supplementary material.

### Determination of the limit of detection

For the methodology established in this study, the limit of detection (LOD) was evaluated via serially diluted preparations of HI synthetic DNA, HI gDNA, and HI bacterial suspensions (all experiments were performed with at least three independent replicates). Following the LOD determination, near-threshold concentrations were selected as test templates for 20 replicate measurements. Subsequently, more accurate LODs were determined through probit regression analysis with a 95% reproducibility probability, which is an established approach for evaluating the reliability of molecular detection methods (25).

### Statistical analysis

The mean ± standard deviation (SD) of fluorescence values from ≥3 independent reactions in this study was used to express the fluorescence signal intensity in the CRISPR assay. GraphPad Prism software (GraphPad, Inc., La Jolla, CA, USA) was used to calculate the means and standard deviations. Statistical analysis was performed via paired t tests to compare both (i) fluorescence differences between conditions within the same group during optimization experiments, and (ii) fluorescence/CT value variations between template concentrations and normal controls during sensitivity assessment experiments. The statistical tests in this study were two-sided, and statistical significance was set at *P* < 0.05. The probabilistic regression analysis was performed with MedCalc 19.0.4 software (MedCalc, Ostend, Belgium). Receiver operating characteristic (ROC) analysis was performed on the detection results via GraphPad Prism (GraphPad, Inc., La Jolla, CA, USA) in the sample validation experiments, and the cutoff values were determined according to Youden’s index.

The PCR/RAA amplification steps, qPCR, ddPCR operation steps, fluorescence-based CRISPR/Cas13a detection steps, lateral flow readout-based CRISPR/Cas13a detection steps, as well as the detailed operation steps of EFORCA can be found in the Supplementary materials.

## RESULTS

### Construction and optimization of the two-step RAA CRISPR assay for HI DNA

We designed multiple crRNA sequences and RAA primers. The results of agarose gel electrophoresis of the amplification products of different primer combinations revealed that the RAA-F2R3 combination was the best pair of RAA primers (Fig. S1A). After confirming the best primer pair, the RAA amplification products were used for CRISPR detection to screen crRNA1-5, and the fluorescence signal values were used to determine that crRNA2 was the best crRNA (Fig. S1C). We established a two-step RAA-CRISPR assay for detecting HI DNA using optimal primers and crRNA sequences. The process was as follows: first, DNA was extracted from the collected clinical samples, and then, RAA was performed, followed by CRISPR/Cas13a detection. When the target is present, it can be recognized by the Cas13a–crRNA complex, triggering Cas protein cleavage activity. The RNA reporter gene in the system is cleaved, and the reporter gene readout is measured with a fluorescence detector (Fig. 1A).

Since both RAA amplification and CRISPR detection involve enzymatic reactions, parameters such as the reaction temperature and DNA template can affect detection efficiency. To improve the detection performance, we optimized the RAA amplification system and CRISPR detection system based on the endpoint fluorescence signal intensity. The results revealed that the optimal conditions for RAA amplification were as follows: 5 µL of amplification template and reaction at 42°C for 30 min (Fig. S2A).

### Assessment of the two-step RAA-CRISPR assay for HI DNA

**Fig 2:**
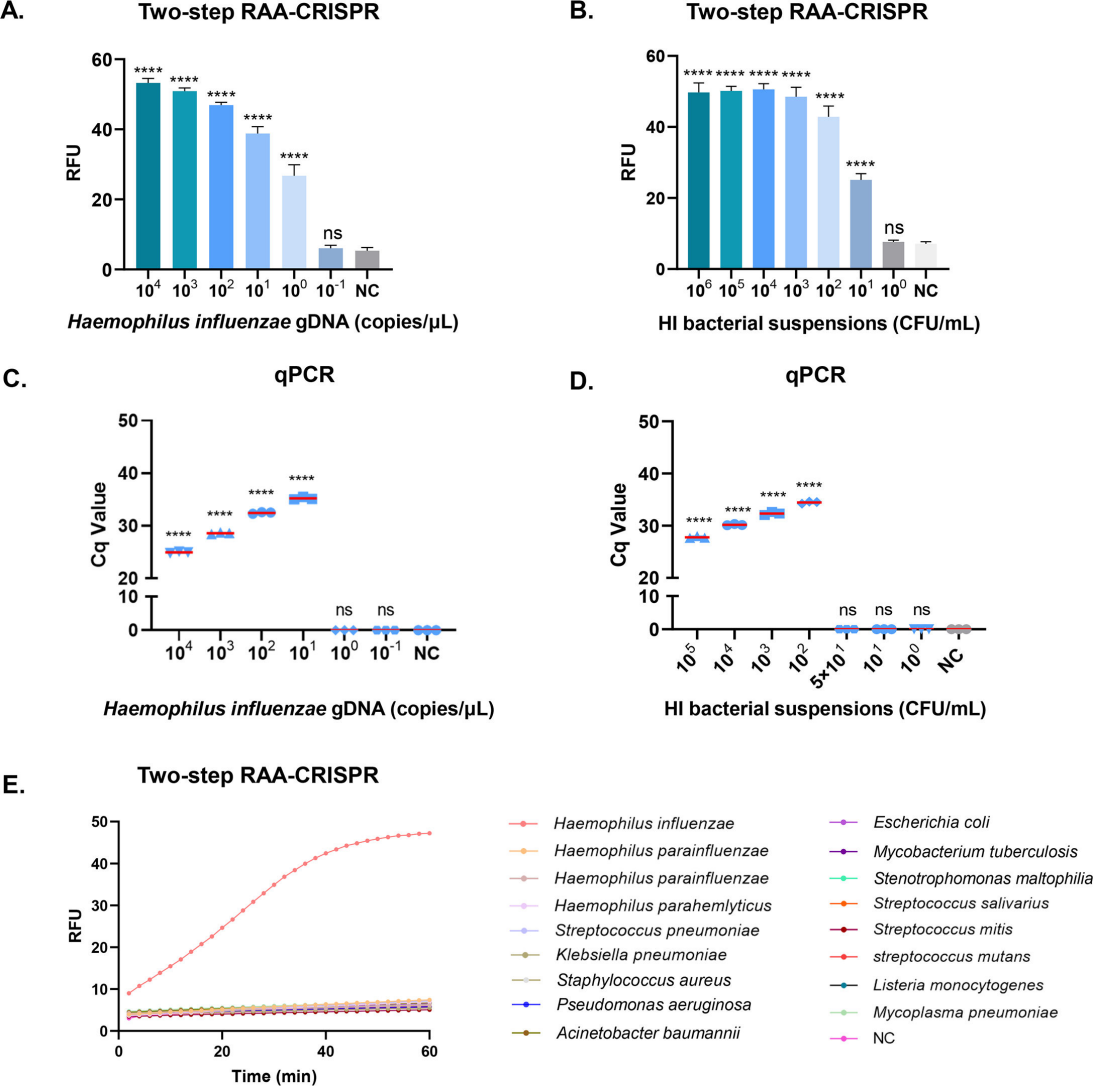
Construction and evaluation of the two-step RAA-CRISPR assay for HI DNA. (**A, B**) Sensitivity evaluation of the two-step RAA-CRISPR assay using gradient dilutions of HI genomic DNA and HI bacterial suspensions. (**C, D**) Sensitivity evaluation of the qPCR using gradient dilutions of HI genomic DNA and HI bacterial suspensions. (**E**) Specificity evaluation of the two-step RAA-CRISPR assay for detecting HI DNA using clinical bacterial strains. The data are representative of at least three independent experiments. NC is the result of a sterile water test without enzymes. RFU, relative fluorescence units.

Next, we evaluated the sensitivity and specificity of the two-step RAA-CRISPR assay. We used gradient-diluted synthetic DNA as the template for detection. The fluorescence results revealed that the sensitivity of the two-step RAA-CRISPR assay reached 1 copy/μL (Fig. S1D). To simulate the real clinical detection scenario more closely, we evaluated the detection performance of the method with a gradient-diluted standard bacterial DNA solution. After the bacterial DNA was extracted, we used ddPCR for quantification, determined the concentration, and then performed gradient dilution and detection (the ddPCR assay results are described in detail in Fig. S3). The fluorescence results revealed that the sensitivity of the two-step RAA-CRISPR assay reached 1 copy/μL for HI gDNA. To obtain a more accurate LOD, we performed probit regression analysis with a 95% probability of reproducibility. Twenty replicate experiments determined the LOD to be 1 copy/μL for gDNA, matching ddPCR sensitivity and surpassing qPCR performance (10 copies/μL) (Fig. 2A and C; Fig. S3A and 4E). Additionally, we evaluated serially diluted bacterial suspensions, and the achieved LOD was 10 CFU/mL. Similarly, 20 replicate experiments determined the LOD to be 10 CFU/mL for bacterial suspensions (consistent with ddPCR), which was lower than the 10^2^ CFU/mL LOD of qPCR (Fig. 2B and D; Fig. S3B and 4E).

Moreover, for the convenience of result interpretation, we adopted a lateral flow strip method. The working principle of the test strip is detailed in the Materials and Methods of the Supplementary information. In the two-step RAA-CRISPR assay, for the HI gDNA and bacterial suspensions with gradient dilutions, the sensitivities of the lateral flow strip method were 1 copy/μL and 10 CFU/mL, respectively (Fig. S5B and C). Similarly, 20 replicate experiments established that the two-step RAA-CRISPR assay with lateral flow readout achieved an LOD of 1.3 copies/μL for gDNA and 11 CFU/mL for bacterial suspensions (Fig. S4E). The results indicated that the sensitivity of the lateral flow strip method was essentially comparable to that of the fluorescence-based method.

Next, we evaluated the specificity of the CRISPR detection method with multiple bacterial pathogens, including *Klebsiella pneumoniae*, *Streptococcus pneumoniae*, *Acinetobacter baumannii*, and *Pseudomonas aeruginosa,* as well as several other pathogens (Table S3). The specificity assessment experiment was repeated three times. The results showed that the two-step RAA-CRISPR assay had good specificity and no cross-reaction with nontargeted strains (Fig. 2E).

### Construction and assessment of the two-step PCR-CRISPR assay for HI DNA

Similarly, we established a two-step PCR-CRISPR assay for detecting HI DNA using the optimal primers (PCR-F3R1) and crRNA sequences (crRNA2) (Fig. 1A; Fig. S1B and C). We also optimized the PCR amplification system based on the endpoint fluorescence signal intensity (Fig. S2B).

Next, we evaluated the sensitivity and specificity of the two-step PCR-CRISPR assay. The fluorescence detection results demonstrated that the two-step PCR-CRISPR assay achieved a sensitivity of 1 copy/μL when either serially diluted synthetic DNA or bacterial gDNA solutions were used as templates (Fig. S1D and S2D). Using gDNA as the detection target, 20 replicate experiments determined the LOD to be 1 copy/μL, matching ddPCR sensitivity and surpassing qPCR performance (10 copies/μL) (Fig. 2C; Fig. S3A and S4E). Similarly, we evaluated serially diluted bacterial suspensions, which achieved a LOD of 10 CFU/mL. Twenty replicate experiments determined the LOD to be 10 CFU/mL for bacterial suspensions (consistent with ddPCR), which was lower than the 10^2^ CFU/mL LOD of qPCR (Fig. 2D; Fig. S2E, S3B, and S4E). The specificity assessment experiment confirmed that the two-step PCR-CRISPR assay also had good specificity and no cross-reaction with nontargeted strains (Fig. S2F).

### Establishment and optimization of the one-pot RAA-CRISPR assay

**Fig 3:**
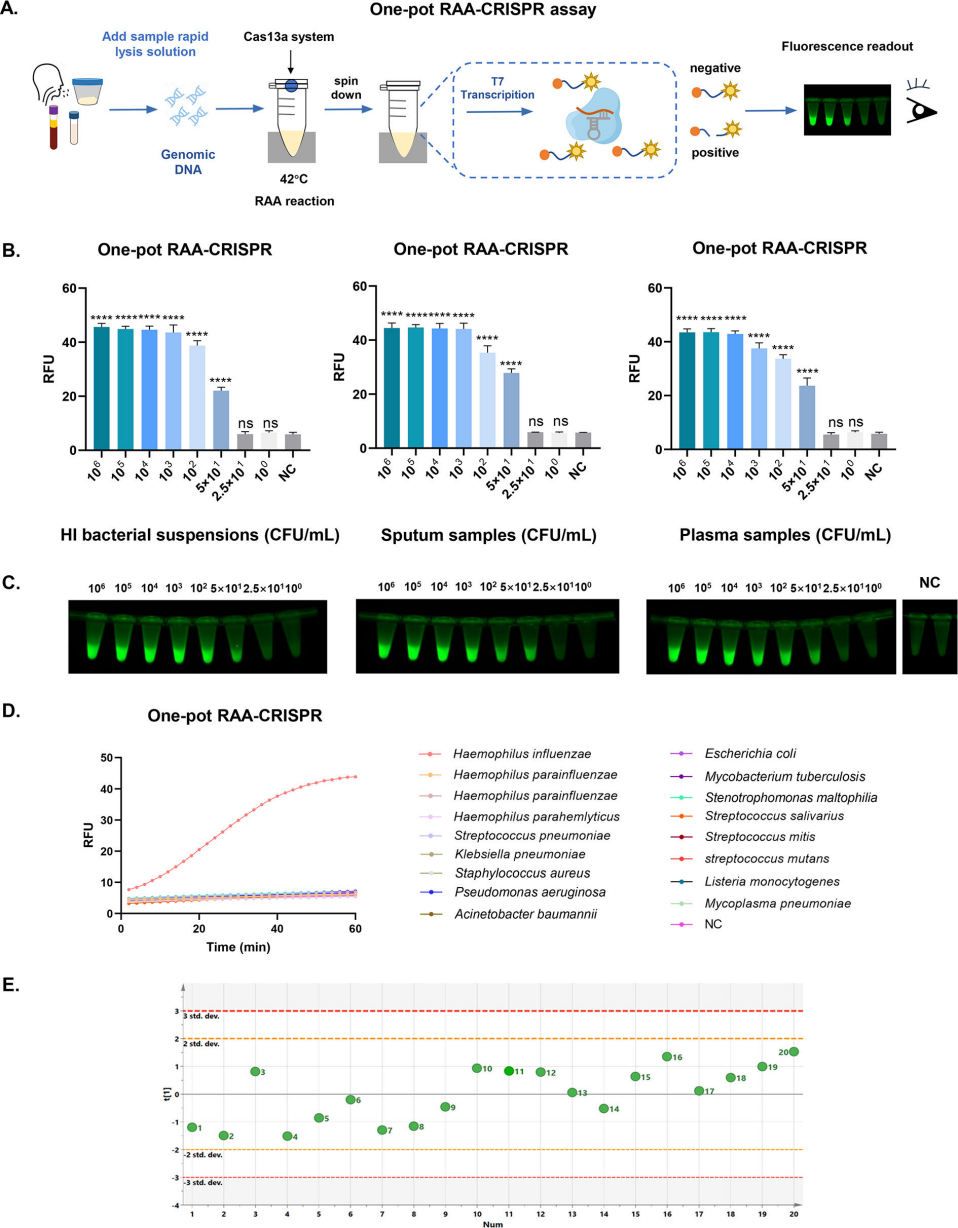
Construction and evaluation of the one-pot RAA-CRISPR assay. (**A**) Schematic diagram of the process of HI detection via one-pot RAA-CRISPR assay. (**B**) LOD of the one-pot RAA-CRISPR assay for HI DNA detection in gradient dilutions of HI bacterial suspensions, simulated sputum, and plasma samples. The data are representative of three independent experiments. (**C**) A map of the test results observed under blue light. (**D**) Specificity evaluation of the one-pot RAA-CRISPR assay using clinical bacterial strains. (**E**) The stability of the one-pot RAA-CRISPR assay was evaluated via 20 replicates of the same template, and the endpoint fluorescence value was analyzed via SIMCA. The data are representative of three independent experiments. ****P < 0.0001. NC, negative control.

Following the establishment of the two-step assay, we began to explore how to achieve point‐of-care testing (POCT) detection. The two-step CRISPR assay has been verified to demonstrate good detection performance, but the procedure is relatively complex and prone to aerosol contamination due to tube-opening operations. Therefore, we aimed to perform the RAA reaction and the CRISPR reaction in the same tube. Meanwhile, rapid sample processing is crucial for harnessing the full utility of CRISPR technology in POCT. Therefore, in this study, we incorporated a rapid lysis step and established a rapid one-pot RAA-CRISPR assay. We added the RAA and CRISPR components to the bottom of the tube and the important CRISPR components to the cap to reduce the interference between the components of the two systems (Fig. 3A).

To achieve better lysis efficiency, we explored and optimized the rapid lysis step using 10^3^ CFU/mL sputum samples and simulated plasma samples. The reason why we did not choose whole blood samples is that we evaluated the detection performance of this method in both whole blood and separate plasma samples. When the HI concentration was 10^3^ CFU/mL, there was a difference in the detection performance between whole blood and separated plasma samples, likely due to the presence of nontarget substances in the blood, including fibers, cells, and free DNA (Fig. S6A and B). The fluorescence results revealed that the optimal ratio of sample to lysate was 1:6, resulting in increased lysis efficiency (Fig. S6C). For the plasma samples, adding lysis solution directly (sample to lysis solution 1:6) before the two-step RAA-CRISPR yielded positive results, whereas for the sputum samples, using 4% NaOH treatment (shaking for 1 min) before adding lysis buffer for the two-step RAA-CRISPR reaction improved the detection efficiency (Fig. S6D). After optimizing the lysis step, we evaluated the lysis performance by the two-step RAA assay (Fig. S7A). The results of the use of gradient-diluted simulated sputum samples and plasma samples for rapid lysis and two-step RAA-CRISPR detection revealed that the sensitivity of the fluorescence and lateral flow strip methods for sputum samples and plasma samples reached 25 CFU/mL. Although rapid sample lysis led to slightly lower sensitivity than nucleic acid extraction, the entire process was convenient and fast (Fig. S7B and C).

Next, we optimized the one-pot RAA-CRISPR assay based on the endpoint fluorescence signal intensity. The results revealed that the optimal conditions were as follows: 10 µL of RAA MIX and reaction at 42°C for 15 min, the Cas13a/crRNA binary complex concentration was 200 mM, and the mixture was reacted at 37°C for 10 min (Fig. S6E and F).

### Assessment of the one-pot RAA-CRISPR assay for HI DNA

Next, we evaluated the one-pot RAA-CRISPR assay using a bacterial suspension. The fluorescence results revealed that the sensitivity reached 50 CFU/mL (fluorescence under blue light showed the same results), and 20 replicate tests established an accurate LOD of 50 CFU/mL (Fig. 3B). Although the LOD was higher than that of ddPCR (10 CFU/mL) and the two-step PCR/RAA-CRISPR assay (10 CFU/mL), it remained lower than qPCR (100 CFU/mL) (Fig. 2B and D; Fig. S3B and S4E). To further evaluate the feasibility of the one-pot RAA-CRISPR assay for detecting HI infection in clinical samples, simulated positive sputum and plasma samples were used. For the simulated clinical samples (sputum and plasma samples), the fluorescence results revealed that the sensitivity reached 50 CFU/mL (fluorescence under blue light showed the same results), 20 replicate tests determined the LOD to be 54 and 56 CFU/mL, respectively (Fig. 3B; Fig. S4C). The specificity evaluation confirmed that the one-pot RAA-CRISPR assay had good specificity and no cross-reaction with nontargeted strains (Fig. 3D). The specificity assessment experiment was repeated three times. Furthermore, the one-step RAA-CRISPR assay demonstrated a coefficient of variation of 2.6% for fluorescence values across 20 replicate tests. The data were uniformly distributed within the range of the mean ± 2 SD, with no significant outliers observed, demonstrating adequate reproducibility of the detection system (Fig. 3E).

### Evaluation of the two-step PCR-CRISPR assay, the two-step RAA-CRISPR assay, and the one-pot assay for detection in simulated sputum and plasma samples

**Fig 4:**
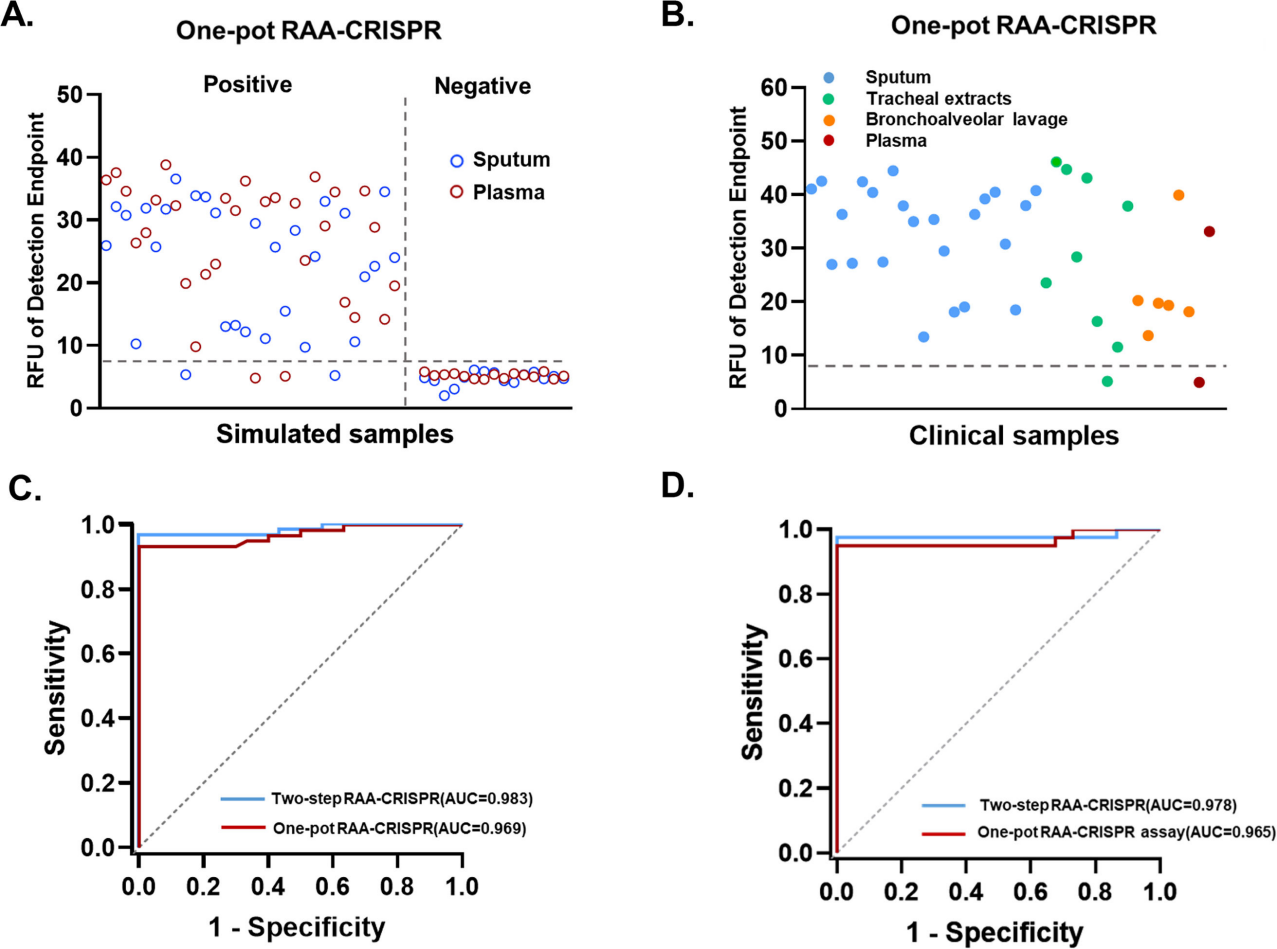
Validation of the CRISPR/Cas13a assay for HI DNA detection. (**A**) The results of the one-pot RAA-CRISPR assay in simulated sputum samples and simulated plasma samples. The simulated samples included 60 positive samples, 30 sputum samples (blue), 30 plasma samples (red), and 30 negative samples (15 sputum samples and 15 plasma samples). (**B**) The results of the one-pot RAA-CRISPR assay for clinical samples, including 23 sputum samples (blue), 9 tracheal extracts (green), 6 bronchoalveolar lavage samples (orange), and 2 plasma samples (red). (**C**) Analysis of the ROC curve from simulated sample testing. The blue lines represent the two-step RAA-CRISPR assay results, and the red lines represent the one-pot RAA-CRISPR assay results. (**D**) Analysis of the ROC curve of the detection results for the clinical samples. The blue lines represent the two-step RAA-CRISPR assay results, and the red lines represent the one-pot RAA-CRISPR assay results. The data are representative of three independent experiments.

Subsequently, 90 simulated samples (60 positive and 30 negative) were tested with the two-step PCR-CRISPR assay, the two-step RAA-CRISPR assay, the one-pot assay, qPCR, and ddPCR, with half being sputum samples and the other half being plasma samples (Fig. 4A; Fig. S8A and B). The area under the curve (AUC) values of the two-step PCR-CRISPR assay, the two-step RAA-CRISPR assay, and the one-pot RAA-CRISPR assay results for the simulated samples were 0.992, 0.983, and 0.969, respectively. The cutoff values were determined based on the fluorescence intensity corresponding to the maximum Youden index, with values of 11.4 a.u. for the two-step PCR-CRISPR assay, 0.983 a.u. for the two-step RAA-CRISPR assay, and 7.927 a.u. for the one-pot RAA-CRISPR assay (Fig. 4C; Fig. S8C). The sensitivities of the two-step PCR-CRISPR assay, the two-step RAA-CRISPR assay, and the one-pot RAA-CRISPR assay were 96.7%, 96.7%, and 93.3%, respectively, and the specificity for all three was 100%. The positive percent agreement (PPA) values were 96.7% (95% CI: 87.5%–99.4%), 96.7% (95% CI: 87.5%–99.4%), and 93.3% (95% CI: 83.0%–97.8%), respectively, and the negative percent agreement (NPA) values were 100% (95% CI: 85.9%–100%) (Fig. S9; Table S4). When qPCR was used to test the simulated samples, two positive sputum samples and three positive plasma samples tested negative. The detection sensitivity in sputum was 93.3%, and that in plasma was 90% (Fig. S9; Table S4). This result was confirmed by ddPCR. These results also demonstrated the superiority of the CRISPR method in detecting low concentrations in samples, indicating that CRISPR detection is a faster and more sensitive alternative to qPCR.

### Establishment and evaluation of the EFORCA

**Fig 5:**
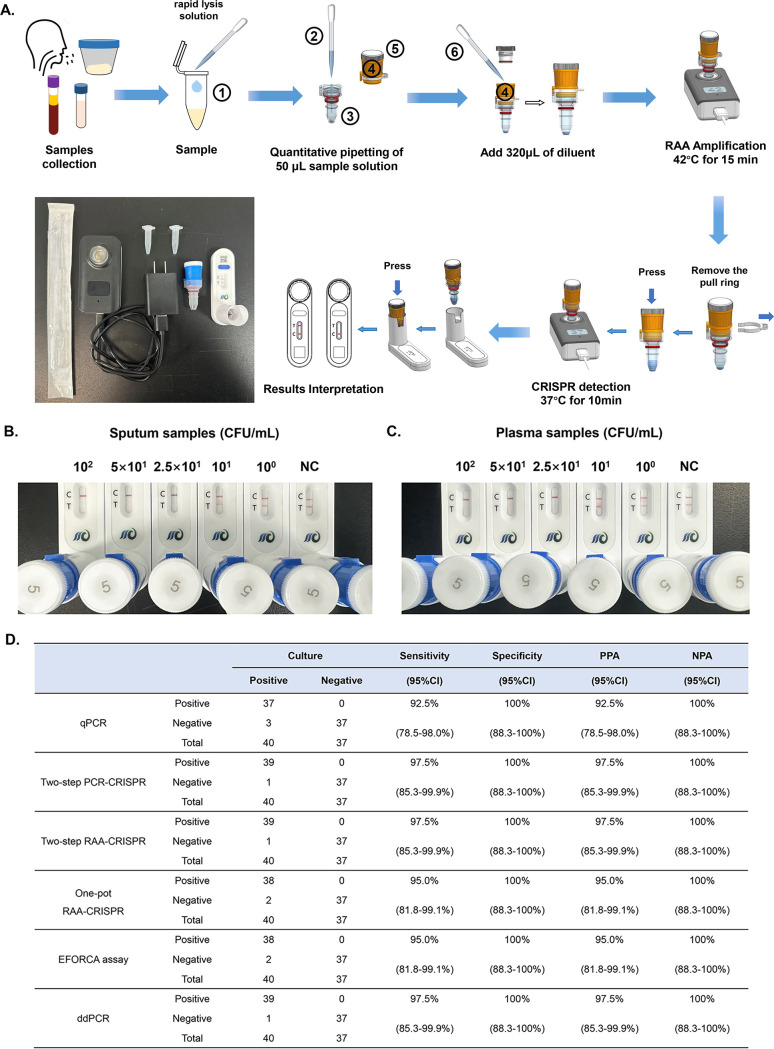
Construction and evaluation of the EFORCA for the detection of HI. (**A**) Schematic diagram of the operation process for the EFORCA device for the detection of HI DNA; the detailed procedures are described in the Supplementary materials. The bottom left picture shows the equipment needed to complete the test. (**B, C**) Results of the EFORCA for serial dilutions of HI-simulated sputum samples and simulated plasma samples (NC: noninfected samples). The data are representative of three independent experiments. (**D**) Comparative analysis of the results of multiple detection methods for clinical samples. The sensitivity, specificity, PPA, and NPA were calculated using Clinical Calculator 1 (http://vassarstats.net/clin1.html#return).

Notably, although the detection sensitivity of the one-pot RAA-CRISPR assay reached 50 CFU/mL, it was somewhat higher than that of the two-step RAA-CRISPR assay (25 CFU/mL). Thus, to ensure optimal detection sensitivity, we designed and fabricated a one-tube device and added freeze-dried reagents, which streamlined the conventional two-step RAA-CRISPR workflow. The detection process only requires lysis buffer, pipettes, and a microincubator to efficiently complete the assay. Moreover, for the convenience of interpretation of the results, we adopted the lateral flow strip method (Fig. 5A). The EFORCA demonstrated a sensitivity of 25 CFU/mL for HI bacterial suspensions (Fig. S10). Consistent results were obtained across 20 independent replicate experiments. Although the LOD was higher than that of ddPCR and the two-step PCR/RAA-CRISPR, it remained lower than one-pot RAA-CRISPR assay and qPCR (Fig. 2B, D, and 3B; Fig. S3B and 4E).

When gradient-diluted simulated sputum samples and plasma samples were subjected to EFORCA, the results revealed that the detection sensitivity for sputum samples and plasma samples reached 25 CFU/mL (Fig. 5B and C). Similarly, 20 replicate tests determined the LOD to be 28 and 25 CFU/ml, respectively. The performance was comparable to the EFORCA for bacterial suspension detection (Fig. S4D and E).

### Comparison of qPCR, the two-step PCR-CRISPR assay, the two-step RAA-CRISPR assay, the one-pot RAA-CRISPR assay, the EFORCA, and ddPCR using clinical samples

Subsequently, 77 clinical samples were tested via qPCR, the two-step PCR-CRISPR assay, the two-step RAA-CRISPR assay, the one-pot RAA-CRISPR assay, the EFORCA, and ddPCR (Fig. 4B; Fig. S8D and E; Table S5). ROC curves were constructed based on the two-step PCR-CRISPR assay, the two-step RAA-CRISPR assay, and the one-pot RAA-CRISPR assay test results. The AUC values for the two-step PCR-CRISPR assay, the two-step RAA-CRISPR assay, and the one-pot RAA-CRISPR assay were 0.994, 0.978, and 0.965, respectively. The cutoff values were set as 10.65 a.u. for the two-step PCR-CRISPR assay, 8.461 a.u. for the two-step RAA-CRISPR assay, and 9.153 a.u. for the one-pot RAA-CRISPR assay (Fig. 4D; Fig. S8F). The sensitivities of the qPCR, two-step PCR-CRISPR, two-step RAA-CRISPR, one-pot RAA-CRISPR, EFORCA, and ddPCR assays were 92.5%, 97.5%, 97.5%, 95%, 95%, and 97.5%, respectively, and the specificities were all 100%. The PPA values were 92.5% (95% CI: 78.5%–98.0%), 97.5% (95% CI: 85.3%–99.9%), 97.5% (95% CI: 85.3%–99.9%), 95% (95% CI: 81.8%–99.1%), 95% (95% CI: 81.8%–99.1%), and 97.5% (95% CI: 85.3%–99.9%), respectively, and the NPA values were all 100% (95% CI: 88.3%–100%) (Fig. 5D; Table S5).

## DISCUSSION

HI is an important human pathogen associated with bacteremia, meningitis, and otitis media (15). In this study, we developed a novel method for detecting HI DNA via CRISPR/Cas13a technology and demonstrated its multiple advantages in diagnosing HI infection. First, our method combines PCR/RAA with CRISPR, ensuring high specificity while achieving a detection sensitivity with a LOD of 1 copy/μL. Second, both the one-pot RAA-CRISPR assay and the EFORCA method integrate RAA with CRISPR, enabling the detection of 50 and 25 CFU/mL HI bacterial suspensions, respectively, within 30 min. We evaluated this method using simulated sputum and plasma samples, and the results revealed high stability and predictive value. Additionally, the EFORCA is simple to perform and portable, effectively addressing some issues in clinical HI infection detection, such as the challenges associated with complex and diverse samples and low detection efficiency. This method is expected to enable on-site detection in small laboratories.

Currently, bacterial culture is a gold standard for HI detection. However, its long turnaround time and stringent culture requirements often result in low detection rates in under-resourced laboratories. MALDI-TOF MS-based methods, which identify bacteria by comparing extracted proteins with universal spectral data, are becoming increasingly popular in microbial diagnosis. However, these tests usually require bacterial culture and identification after relatively pure colonies are obtained, and they are complex, time-consuming, and dependent on professional technicians and expensive instruments, which limits their wide application in small- and medium-sized clinical laboratories. The method developed here enables direct detection from raw samples through rapid lysis and CRISPR-based analysis. This culture-independent approach is simple, fast, and requires just 30 min to achieve highly sensitive and specific results.

Molecular detection techniques, such as qPCR, do not rely on bacterial culture and can be used for direct detection in clinical samples. However, these methods require standard curves to be established for result interpretation. Moreover, these methods may fail to detect low-concentration targets accurately. In this study, we compared qPCR and our method using clinical and simulated samples. For the simulated samples, the sensitivities of the two-step RAA-CRISPR and one-pot RAA-CRISPR assays were 96.7% and 93.3%, respectively, which were greater than that of the qPCR method (91.7%). This finding indicates that our method has better detection performance. For the clinical samples, the sensitivities of the one-pot RAA-CRISPR assay (95.0%) and the EFORCA (95.0%) were lower than those of ddPCR (97.5%) and the two-step PCR/RAA-CRISPR assay (97.5%) but higher than that of qPCR (92.5%). Among the tested samples, one culture-positive specimen yielded negative results in qPCR, the two-step PCR/RAA-CRISPR, the one-pot RAA-CRISPR, the EFORCA, and the ddPCR assays. We speculate that this result may be caused by the excessively low concentration of the target in the sample, leading to a false-negative nucleic acid test result (Fig. 5D; Table S5). Furthermore, our developed methods offer cost benefits ($1.5 for one-pot RAA-CRISPR and $3.5 for EFORCA) compared to qPCR-based detection. These costs are expected to decrease further with scaled-up manufacturing.

The CRISPR/Cas system has been used as an efficient detection tool for various pathogens (26–28). In our previous research, we demonstrated that the LOD of the two-step RAA-CRISPR assay for detecting *Klebsiella pneumoniae* gDNA was 1 copy/μL, which was also verified in clinical strain samples (29). In this study, based on the established two-step RAA-CRISPR assay, we attempted to achieve one-pot RAA-CRISPR detection by optimizing the RAA and CRISPR systems and adding some CRISPR components to the tube cap. After evaluation, the LOD was 50 CFU/mL, which was slightly higher than that of the routine CRISPR method. We speculated that the mixture of RAA and CRISPR reagents might have affected the RAA efficiency. Therefore, we designed and fabricated a low-cost, miniaturized one-pot device that enables RAA and CRISPR reactions to be performed in separate chambers for amplification, followed by a simple mixing operation to complete the detection. This method simplifies the procedure and effectively prevents aerosol contamination to ensure the accuracy of the detection results. Moreover, a rapid lysis step was added to reduce the sample processing time (Fig. S11). Previous studies have established a one-pot method termed ExCad by combining LAMP and Cas12b for detecting *Streptococcus pneumoniae*. However, the absence of lyophilization and a dedicated device in that method hindered its advancement for POCT applications (30). In this study, to ensure the stability of detection and reduce the impact of reagent transport and storage conditions on detection, we freeze-dried the components of the RAA and CRISPR reaction systems. This allows the assay to be performed simply by adding buffer to reconstitute the reagents. Consequently, the EFORCA overcomes the challenges faced in previous studies (29, 31), including the inability to process samples quickly, the inability to perform one-tube detection, dependence on specialized operators, and difficulties in reagent/transport or storage, and realizes the feasibility of applying CRISPR technology for POCT.

Our study has several limitations. First, the rapid lysis step for the sample needs further optimization. The detection results for serial dilutions of simulated positive samples revealed that the sensitivity obtained with the rapid sample lysis method was lower than that obtained with nucleic acid extraction. The method of nucleic acid extraction can directly affect the results of detection. In this study, we found that pretreatment with 4% NaOH prior to lysis buffer addition in the two-step RAA-CRISPR assay significantly improved detection efficiency. This improvement may be attributed to the sputum’s high mucin content, which encapsulates both microorganisms and nucleic acids. Treatment with 4% NaOH effectively reduces sputum viscosity, thereby enhancing nucleic acid lysis efficiency. These findings suggest that optimizing the sample pretreatment process may be an effective approach to enhance lysis efficiency. In future research, we will continue to explore the rapid lysis of samples to improve the detection performance. In addition, the CRISPR/Cas13a assay is unable to quantify HI DNA because the incidental cleavage activity of Cas13a is “random.” Further studies are needed to address this issue.

In summary, this study developed sensitive two-step PCR-CRISPR and RAA-CRISPR assays, then engineered a one-pot RAA-CRISPR assay by integrating rapid lysis with RAA and CRISPR technologies for HI detection. Additionally, reagent lyophilization, rapid lysis technology, and a one-pot detection device were integrated to construct the EFORCA. This assay, which is sensitive, specific, rapid, and easy to perform, is expected to be a powerful tool for detecting HI infection in small laboratories.

## Data Availability

All the data are available in the main text or the supplemental materials.

## SUPPLEMENTAL MATERIAL

Supplemental figures and tables jcm.00535-25-s0001.docx Fig. S1 to S11; Tables S1 to S5.

ASM does not own the copyrights to Supplemental Material that may be linked to, or accessed through, an article. The authors have granted ASM a non-exclusive, world-wide license to publish the Supplemental Material files. Please contact the corresponding author directly for reuse.
