# Supplementary material for: An extraction-free, lyophilized one-pot RAA-CRISPR assay for point-of-care testing of Haemophilus influenzae
Source: J Clin Microbiol. 2025 Sep 23;63(11):e00535-25. doi: 10.1128/jcm.00535-25 (PMC12607747; doi:10.1128/jcm.00535-25)
Supplement: Supplemental figures and tables — Fig. S1 to S11; Tables S1 to S5. [file jcm.00535-25-s0001.docx]

**Supplementary materials and methods**

**Purification of crRNAs**

PCR primers were designed and synthesized for the crRNA DNA, and the primers included the T7 promoter. The crRNA was generated overnight at 37°C via the HiScribe T7 Quick High Yield RNA Synthesis Kit (New England Biolabs, Inc., Ipswich, MA, USA). The crRNA concentration was measured using the NanoDrop One (Thermo Fisher Scientific, Waltham, MA, USA) after purification using magnetic beads, after which it was diluted to 100 ng/μL for use. The above procedure was performed using RNA-free consumables to ensure purity.

**PCR and RAA**

The PCR system volume was 25 μL, containing 2 μL (1 mM) of forward and reverse primers and 14.5 μL of PCR mix (Biomed), with DNase/RNase-free water added to achieve a final volume of 25 μL. The PCR conditions were as follows: 95°C for 5 min; 30 cycles of 95°C for 30 s, 55°C for 30 s, and 72°C for 50 s; and a final extension step of 72°C for 5 min.

The RAA kit used was provided by Hang Zhou ZC Biotechnology Co., Ltd. (Hangzhou, China). The final total volume was 50 μL, including 2 μL of F (10 mM), 2 μL of R (10 mM) and MgCl_2_. The reaction was performed at 37°C for 30 min, and the negative control was nuclease-free water. An RAA kit (Hangzhou Zhongce Bio-Sci & Tech Co., Ltd., Hangzhou China) was used in this study. Each dry powder tube was a reaction unit to which 25 μL of Buffer A, 2 μL of F (10 mM), 2 μL of R (10 mM) and MgCl_2_ were added, and the mixture was placed in a metal bath at 42°C for 30 min.

**DNA agarose gel electrophoresis**

The 1.5% agarose gel was prepared. Agarose (1.5 g) was mixed with 100 mL of 1XAE solution (Beijing Biomed), heated in a microwave until dissolved, placed at room temperature and cooled to 50 to 60°C, after which 7 μL of GoldenView TM nucleic acid stain (Beijing Biomed) was added. The prepared gel was placed in buffer containing 1X TAE, and 5 μL of amplification product and 1 μL of 6X loading buffer (Beijing Biomed) were added to each well to confirm the position via the BM2000 DNA Marker (Beijing Biomed). After electrophoresis at 160 V for approximately 25 min, the gel block was removed and placed on a gel imager (Bio-Rad, Inc., Hercules, CA, USA) for observation.

**Fluorescence-based CRISPR/Cas13a assays**

The CRISPR system volume was 25 μL, containing 1.5 μL of crRNA (100 ng/μL), 1 μL of Cas13a (GenScript Biotechnology Co., Ltd.), 0.5 μL of T7 RNA polymerase (50000 U/mL, New England Biolabs), 1 μL of RNase inhibitor (40000 U/mL, New England Biolabs), 2 mM NTP mix (10 mM, New England Biolabs), 125 nM quenched fluorescent RNA reporter solution (2,000 nM, RNase Alert, Thermo Fisher Scientific), 0.25 µL of MgCl_2_ solution (1 M), 0.5 μL of HEPES buffer (1 M, Thermo Fisher Scientific), 5 μL of target nucleic acid, and 10.75 μL of RNase-free water. The components were mixed and reacted at 37°C for 1 h, and the fluorescence signals were recorded every 2 min on a Light Cycler® 480 System (Hoffmann‒La Roche). Fluorescence was observed by naked eye using a Chemi Doc XP imager (Bio-Rad, Inc., Hercules, CA, USA) with selected blue light [25].

**Lateral flow readout-based CRISPR/Cas13a assays**

The structure and detection principle of the lateral flow strip: The test line was pre-coated with avidin, while the control line was coated with an anti-FAM antibody. The products were purchased from Hangzhou ZC Biotechnology Co., Ltd. (Hang zhou, China). In the presence of the target DNA, the Cas effector protein’s cleavage activity is triggered, cleaving the reporter molecule. The released biotin is captured on the test line, while the remaining reporter molecule flows to the control line, where it binds to the anti-FAM antibody, generating a colorimetric signal. In the absence of the target DNA, the reporter molecule is captured by avidin on the test line, and the anti-FAM antibody still binds the reporter on the control line, producing signals on both lines (Supplementary Figure 5 A) [2].

The total system volume for the CRISPR reaction was 50 μL, containing 5μL of RAA amplicon, 3 μL of crRNA, 25 nM Cas13a (ZC Biotech Technology Co.), 1 μL of T7 RNA polymerase (50,000 U/mL, New England Biolabs), 1 μL of RNase inhibitor (40,000 U/mL, New England Biolabs), 2 mM NTP mix (10 mM, New England Biolabs), 2 nM reporter RNA (5'/6-FAM/UUUUUUUUUUUUUUUUUUUU-Bio/3'), 0.5 µL of MgCl_2_ solution (1 M), 1 μL of HEPES buffer (1 M, Thermo Fisher Scientific), and 26.5 μL of RNase-free water. The components were mixed and placed in a metal bath for 30 min at 37°C for the reaction.

**Quantitative Rea-ltime-PCR (qPCR)**

The instrument used for these experiments was LightCycler­® 480 Instrument II. The total volume of the reaction was 20μL, including 10μL Fast Advanced Master Mix (Thermo Fisher Scientific, Waltham, MA, USA), 5μL of DNA template, 0.5 μL of the 10 nM primer and probe and 2.5μL of DNase/RNase-free water. The qPCR conditions were as follows: 94°C for 3 min, followed by 94°C for 30 s (denaturation) and 58°C for 45s (annealing) for 40 cycles.

**Droplet Digital PCR (ddPCR)**

The total system volume was 30μL, including 5μL of DNA template, 7.5μL of ddPCR Supermix (no dUTP), 0.9μL of probe, 2.4μL of 10 mM F/R and 0.4μL of DNase/RNase-free water, the reaction mixture was converted to droplets using a droplet generator. The reaction conditions were as follows: 95°C for 10 min, denaturation at 94°C for 30 s, annealing at 60°C for 1 min and 39 cycles. The tubes containing the amplification products were then transferred to a droplet detection chip and read via a QX200 reader for fluorescence detection. ddPCR was performed via a Droplet Digital TM PCR machine (Xin Yi Biotechnology Co).

**One-pot assay procedure**

1. Sample processing

Plasma samples: lysis solution was added to the plasma samples (the ratio of sample to lysate was 1:6), which were then mixed and allowed to stand for 1 min before detection.

Sputum samples: sputum samples were mixed with 4% NaOH (the sample-to-4% NaOH ratio was 1:1), mixed and shaken for 1 min, followed by addition of the lysis solution, after which the samples were mixed and allowed to stand for 1 min (the sample-to-lysis solution ratio was 1:6).

2. Addition of sample to be tested

2.1 The device was removed; tube 3 was kept vertical and carefully opened (the "T "slot to break apart) (Figure 5A).

2.2 Fifty microliters of the lysed sample were added into tube 3 with a dosing pipette and mixed thoroughly, ensuring that the liquid was at the bottom (the mixing step was very important here).

3. The tube cap was carefully unscrewed, and 320 μL of diluent was carefully added using a dosing pipette. The lid was then closed [1].

4. Isothermal amplification and detection


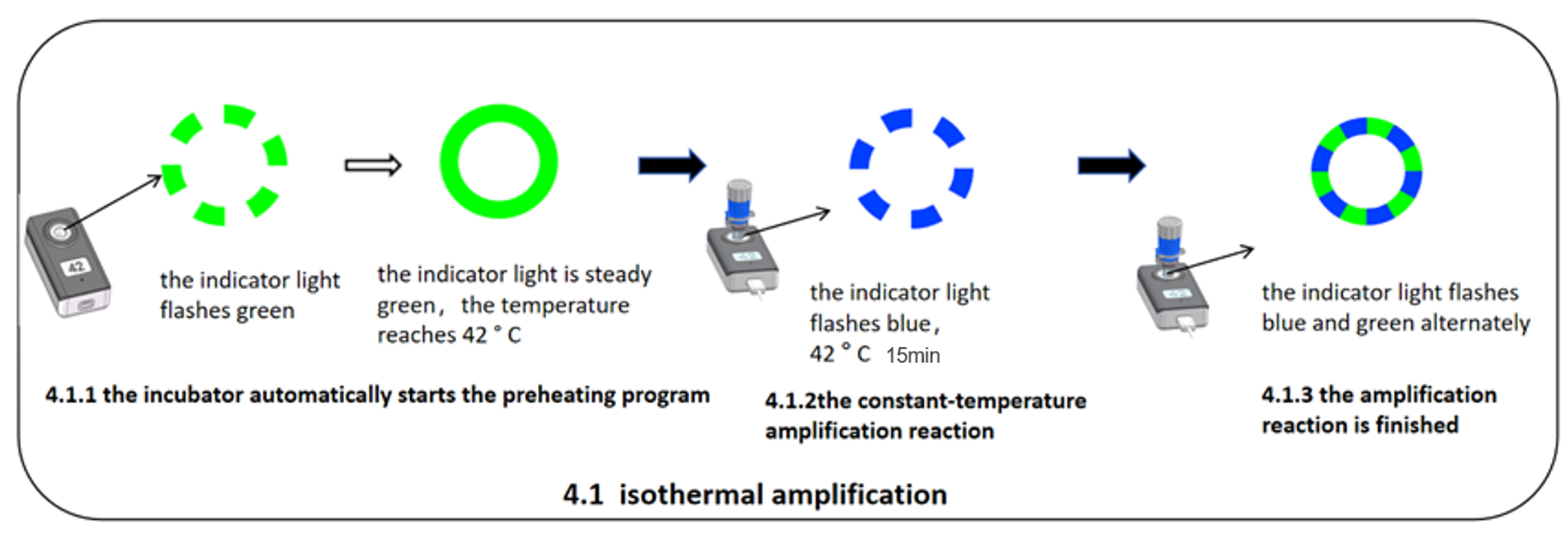


4.1.1 A flashing green indicator light on the small heating device indicated that preheating was complete, while a flashing green indicator light (
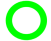
) indicated that the temperature had reached 42°C.

4.1.2 The liquid in tube 3 was placed at the bottom of the heating device, and the constant temperature was maintained for an additional 15 min, the indicator light continued to flash blue light (
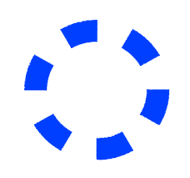
).

4.1.3 After amplification, the device played an alarm, and the indicator light emitted alternating blue‒green flashes (
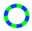
).

4.2 CRISPR detection


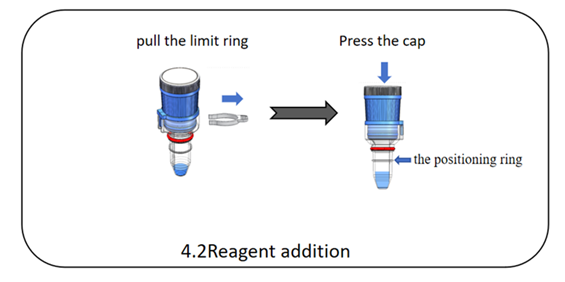


4.2.1 The ring between tube 3 and tube 4 was pulled, placed vertically and pressed from the top until all the liquid in tube 4 entered tube 3, ensuring that the liquid was at the bottom of the tube after thorough mixing (this step was critical).

Note: The liquid level of the reaction tube was monitored. If it had not reached the middle position of the positioning ring, the device was held by hand (with tube no. 3 below and tube no. 5 above) and shaken to ensure that the liquid level reached the required position.

4.3 Nucleic acid testing


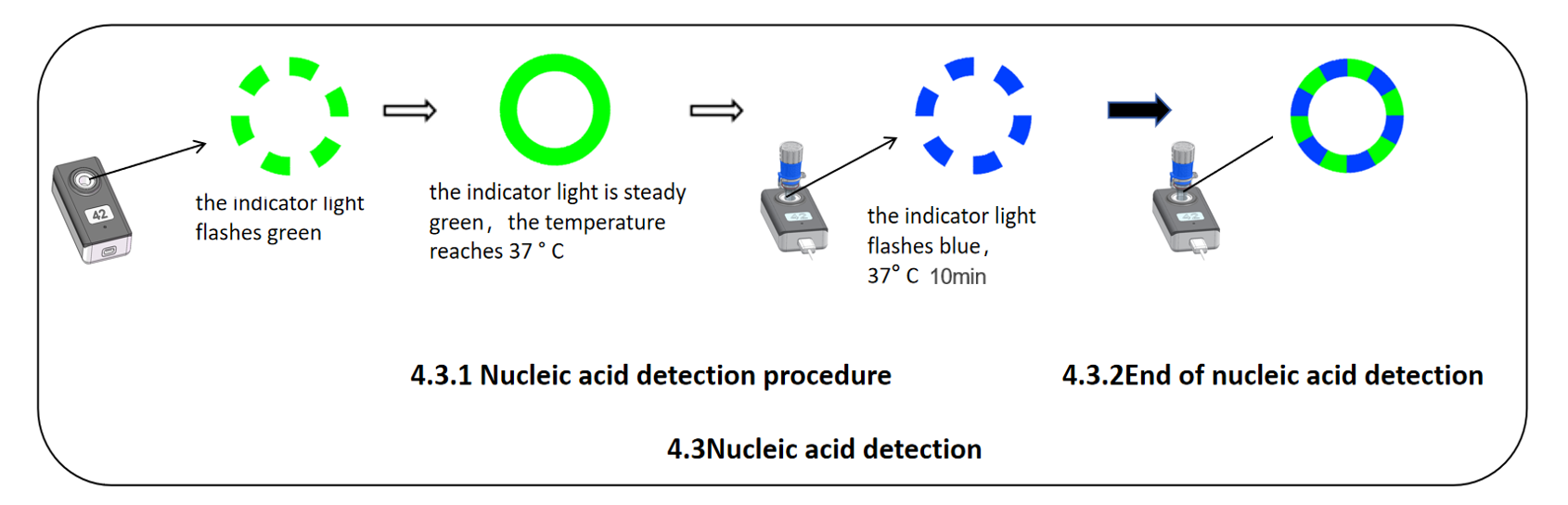


4.3.1 The incubator was allowed to cool automatically to 37°C (i.e., until the indicator light was green
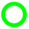
). The reaction tube was placed in a small incubator (ensuring that the liquid was at the bottom of the tube), and the constant temperature was maintained for an additional 10 min (the indicator light continued to flash blue light
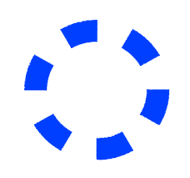
).

4.3.2 After the reaction, the device emitted an alarm sound (and the indicator light flashed alternately blue and green
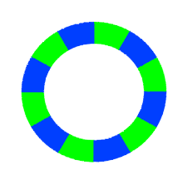
).

5. The test strip was used to read the results.


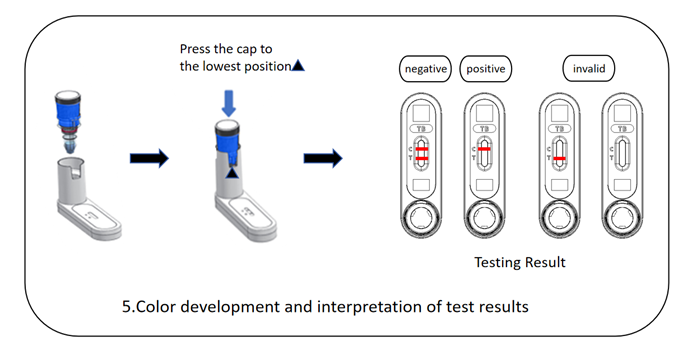


After the reaction, the mixture was gently shaken, the reaction tube was inserted vertically into the test strip device, and the test results were observed for 5 min.

**HI synthetic DNA sequence:**

GACCAACGGATTAAAAGTCCGCTGCTCTACCGACTGAGCTAACGACCCACTGTGTTAATGGATTTATGATAACGCATTTGTTCTAGAAGCAAAAGAATAAATTCATTCTTTCAAGCTAATTGGTTAAATTACAAACGATGAAAACAAAACAGGATCCAATAAAAAATGGATCCTGTTTTTCAAGTATTAGAAATACTAAGAATTAGTACGCTAACACTGCACGACGGTTTTTAGAATATGCAGCTTCATCATGACCTAATACTGCAGGTTTTTCTTCACCGTAAGATACTGTGCCTAATTTACCAGCATCAACACCTTTACCAGCTAAATAACCTTTAACTGCATCTGCACGACGTTGGCCTAATGCGATGTTGTATTCTGGTGTACCACGTTCATCAGTGTTACCTTCTACTAATACTTTAGCAGCTGGCGTTGCATTTAAATATGCAGCGTGTGCATCTAAGATTTGAACGTATTCACCAGTAATGTCATATTTATCAAAACCGAAATAAACGGTATTGTAACGTTGTTGAAGATCAGCAACAGAGTAACCGCCAAAAGTTTGAGCAGCACCATTGCCTGCAGCATCGTTGTTAGATGAACTACAAGCTGCTAATGCAGCTACAGAACCTGCAACTAATAATGATTT

**Supplementary Table1.** Sequences of HI PCR, RAA and qPCR primers.

| Name | Sequences |
| --- | --- |
| PCR-F1 | 5’-CGACGGTTTTTAGAATATGCAGCT-3’ |
| PCR-R1 | 5’-GAACGTGGTACACCAGAATAC-3’ |
| PCR-F2 | 5’-ACGGTTTTTAGAATATGCAGCTTCA-3’ |
| PCR-R2 | 5’-GTGAATACGTTCAAATCTTAG-3’ |
| PCR-F3 | 5’-ATGCAGCTTCATCRTGACCT-3’ |
| PCR-R3 | 5’- TGCTGATCTTCAACAACGTTACA -3’ |
| RAA-F1 | 5’-CCTAATACTGCAGGTTTTTCTTCACCGTAA-3’ |
| RAA-R1 | 5’-ACCAGAATACAACATCGCATTAGGCCAACG-3’ |
| RAA-F2 | 5’-TAATACTGCAGGTTTTTCTTCACCGTAAGA-3’ |
| RAA-R2 | 5’-ACTGAYGAACGTGGTACACCAGAATACAAC-3’ |
| RAA-F3 | 5’-TGCAGGTTTTTCTTCACCGTAAGATACYGT-3’ |
| RAA-R3 | 5’-TGAYGAACGTGGTACACCAGAATACAACAT-3’ |
| qPCR-F | 5’-CAACGCCAGCTGCTAAAGTA-3’ |
| qPCR-R | 5’-CAGCATCAACACCTTTACCAGC-3’ |
| qPCR-probe | 5’-FAM-CAACGTCGTGCAGATGC-BHQ1-3’ |

**Supplementary Table2.** HI crRNA sequences.

| Name | Sequences (5’- 3’) |
| --- | --- |
| RAA-crRNA1 | GGGAUUUAGACUACCCCAAAAACGAAGGGGACUAAAACGCCUAAUUUACCAGCAUCAACACCUUUA |
| RAA-crRNA2 | GGGAUUUAGACUACCCCAAAAACGAAGGGGACUAAAACCCUAAUUUACCAGCAUCAACACCUUUAC |
| RAA-crRNA3 | GGGAUUUAGACUACCCCAAAAACGAAGGGGACUAAAACCUAAUUUACCAGCAUCAACACCUUUACC |
| RAA-crRNA4 | GGGAUUUAGACUACCCCAAAAACGAAGGGGACUAAAACUAAUUUACCAGCAUCAACACCUUUACCA |
| RAA-crRNA5 | GGGAUUUAGACUACCCCAAAAACGAAGGGGACUAAAACAAUUUACCAGCAUCAACACCUUUACCAG |

**Supplementary Table 3.** Strains used in this study.

| Strain | Type (Strain ID) | No. of strains | Detection result |
| --- | --- | --- | --- |
| *Haemophilus influenzae* | Reference strain (ATCC 49247) | 1 | P |
| *Haemophilus parainfluenzae* | Isolated strain | 1 | N |
| *Haemophilus hemolyticus* | Isolated strain | 1 | N |
| *Haemophilus parahemlyticus* | Isolated strain | 1 | N |
| *Streptococcus pneumoniae* | Reference strain (ATCC 49619) | 1 | N |
| *Klebsiella pneumoniae* | Reference strain (ATCC 700603) | 1 | N |
| *Staphylococcus aureus* | Reference strain (ATCC 29213) | 1 | N |
| *Pseudomonas aeruginosa* | Reference strain (ATCC 27853) | 1 | N |
| *Acinetobacter baumannii* | Isolated strain | 1 | N |
| *Escherichia coli* | Reference strain (ATCC 25922) | 1 | N |
| *Mycobacterium tuberculosis* | Reference strain (H37Rv) | 1 | N |
| *Stenotrophomonas maltophilia* | Isolated strain | 1 | N |
| *Streptococcus salivarius* | Isolated strain | 1 | N |
| *Streptococcus mitis* | Isolated strain | 1 | N |
| *streptococcus mutans* | Isolated strain | 1 | N |
| *Listeria monocytogenes* | Isolated strain | 1 | N |
| *Mycoplasma pneumoniae* | Reference strain (ATCC15531) | 1 | N |

**Supplementary Table 4.** Results of simulated clinical samples.

| **Sample**  **Number** | **HI-ddPCR （copies/μL）** | **qPCR**  **(Cq value)** | **PCR-CRISPR** | **RAA-CRISPR** | **One-pot assay** |
| --- | --- | --- | --- | --- | --- |
| 1 | 40613.4 | 23.45 | + | + | + |
| 2 | 7748.8 | 25.34 | + | + | + |
| 3 | 820.1 | 29.08 | + | + | + |
| 4 | 12763.2 | 25.92 | + | + | + |
| 5 | 75798.3 | 21.24 | + | + | + |
| 6 | 20518.1 | 23.77 | + | + | + |
| 7 | 3742.5 | 26.32 | + | + | + |
| 8 | 840 | 28.32 | + | + | + |
| 9 | 1.8 | Undetectable | Negative | Negative | Negative |
| 10 | 4607.4 | 27.59 | + | + | + |
| 11 | 89502.7 | 23.31 | + | + | + |
| 12 | 60263 | 24.07 | + | + | + |
| 13 | 114 | 34.58 | + | + | + |
| 14 | 5076.4 | 25.45 | + | + | + |
| 15 | 65067.7 | 23.17 | + | + | + |
| 16 | 4095.3 | 25.16 | + | + | + |
| 17 | 15713.3 | 25.82 | + | + | + |
| 18 | 116.8 | 34.8 | + | + | + |
| 19 | 60664.6 | 22.46 | + | + | + |
| 20 | 81 | 35.34 | + | + | + |
| 21 | 820.2 | 31.08 | + | + | + |
| 22 | 58642.5 | 22.92 | + | + | + |
| 23 | 111850 | 23.21 | + | + | + |
| 24 | 31 | Undetectable | + | + | Negative |
| 25 | 128.9 | 33.75 | + | + | + |
| 26 | 580 | 29.58 | + | + | + |
| 27 | 27932.9 | 24.99 | + | + | + |
| 28 | 7911.4 | 26.81 | + | + | + |
| 29 | 16043.5 | 23.79 | + | + | + |
| 30 | 126692.2 | 22.12 | + | + | + |
| 31 | 418.7 | 33.75 | + | + | + |
| 32 | 51051.3 | 22.12 | + | + | + |
| 33 | 7695.1 | 25.85 | + | + | + |
| 34 | 592.1 | 28.55 | + | + | + |
| 35 | 374 | 33.21 | + | + | + |
| 36 | 32988.5 | 23.7 | + | + | + |
| 37 | 10153.7 | 25.45 | + | + | + |
| 38 | 127589.1 | 20.87 | + | + | + |
| 39 | 5555.6 | 26.32 | + | + | + |
| 40 | 198.1 | 34.83 | + | + | + |
| 41 | 2956.7 | 28.23 | + | + | + |
| 42 | 274.3 | 31.66 | + | + | + |
| 43 | 2797.2 | 26.31 | + | + | + |
| 44 | 922.7 | 30.41 | + | + | + |
| 45 | 321.8 | 28.43 | + | + | + |
| 46 | 0 | Undetectable | Negative | Negative | Negative |
| 47 | 1226 | 26.5 | + | + | + |
| 48 | 1937.2 | 26.4 | + | + | + |
| 49 | 18.8 | Undetectable | + | + | Negative |
| 50 | 655 | 29.02 | + | + | + |
| 51 | 43458.9 | 22.67 | + | + | + |
| 52 | 2573.9 | 29.43 | + | + | + |
| 53 | 830 | 27.01 | + | + | + |
| 54 | 2797.2 | 28.31 | + | + | + |
| 55 | 398.6 | 33.18 | + | + | + |
| 56 | 12941.4 | 26.1 | + | + | + |
| 57 | 302 | 35.02 | + | + | + |
| 58 | 2932.3 | 25.42 | + | + | + |
| 59 | 29.4 | Undetectable | + | + | + |
| 60 | 203.2 | 33.32 | + | + | + |
| 61 | 0 | Undetectable | Negative | Negative | Negative |
| 62 | 0 | Undetectable | Negative | Negative | Negative |
| 63 | 0 | Undetectable | Negative | Negative | Negative |
| 64 | 0 | Undetectable | Negative | Negative | Negative |
| 65 | 0 | Undetectable | Negative | Negative | Negative |
| 66 | 0 | Undetectable | Negative | Negative | Negative |
| 67 | 0 | Undetectable | Negative | Negative | Negative |
| 68 | 0 | Undetectable | Negative | Negative | Negative |
| 69 | 0 | Undetectable | Negative | Negative | Negative |
| 70 | 0 | Undetectable | Negative | Negative | Negative |
| 71 | 0 | Undetectable | Negative | Negative | Negative |
| 72 | 0 | Undetectable | Negative | Negative | Negative |
| 73 | 0 | Undetectable | Negative | Negative | Negative |
| 74 | 0 | Undetectable | Negative | Negative | Negative |
| 75 | 0 | Undetectable | Negative | Negative | Negative |
| 76 | 0 | Undetectable | Negative | Negative | Negative |
| 77 | 0 | Undetectable | Negative | Negative | Negative |
| 78 | 0 | Undetectable | Negative | Negative | Negative |
| 79 | 0 | Undetectable | Negative | Negative | Negative |
| 80 | 0 | Undetectable | Negative | Negative | Negative |
| 81 | 0 | Undetectable | Negative | Negative | Negative |
| 82 | 0 | Undetectable | Negative | Negative | Negative |
| 83 | 0 | Undetectable | Negative | Negative | Negative |
| 84 | 0 | Undetectable | Negative | Negative | Negative |
| 85 | 0 | Undetectable | Negative | Negative | Negative |
| 86 | 0 | Undetectable | Negative | Negative | Negative |
| 87 | 0 | Undetectable | Negative | Negative | Negative |
| 88 | 0 | Undetectable | Negative | Negative | Negative |
| 89 | 0 | Undetectable | Negative | Negative | Negative |
| 90 | 0 | Undetectable | Negative | Negative | Negative |

The first 30 were simulated sputum samples and the last 30 were simulated plasma samples.

**Supplementary Table 5.** Results of clinical samples.

| **Sample Number** | **HI-ddPCR (copies/μL)** | **qPCR** | **PCR-CRISPR** | **RAA-CRISPR** | **One-pot assay** | **EFORCA Assay** |
| --- | --- | --- | --- | --- | --- | --- |
| 1 | 2193.6 | 27.9 | + | + | + | + |
| 2 | 15572 | 23.87 | + | + | + | + |
| 3 | 4549.1 | 26.69 | + | + | + | + |
| 4 | 139 | 35.02 | + | + | + | + |
| 5 | 205011.8 | 20.03 | + | + | + | + |
| 6 | 1160.3 | 30.65 | + | + | + | + |
| 7 | 624.1 | 32.75 | + | + | + | + |
| 8 | 2453.3 | 27.62 | + | + | + | + |
| 9 | 66336.7 | 22.77 | + | + | + | + |
| 10 | 893.7 | 30.58 | + | + | + | + |
| 11 | 37629.3 | 24.95 | + | + | + | + |
| 12 | 132.5 | 34.95 | + | + | + | + |
| 13 | 83156.6 | 23.94 | + | + | + | + |
| 14 | 6486.1 | 27.05 | + | + | + | + |
| 15 | 56902.4 | 21.78 | + | + | + | + |
| 16 | 5237.7 | 28.21 | + | + | + | + |
| 17 | 416.7 | 31.7 | + | + | + | + |
| 18 | 1375.2 | 28.43 | + | + | + | + |
| 19 | 2090.6 | 29.68 | + | + | + | + |
| 20 | 6502.9 | 27.975 | + | + | + | + |
| 21 | 25865.5 | 24.065 | + | + | + | + |
| 22 | 10543.2 | 26.11 | + | + | + | + |
| 23 | 840.3 | 30.665 | + | + | + | + |
| 24 | 151.8 | 34.835 | + | + | + | + |
| 25 | 12414.8 | 23.385 | + | + | + | + |
| 26 | 1638 | 29.28 | + | + | + | + |
| 27 | 594.1 | 32.34 | + | + | + | + |
| 28 | 24336.4 | 22.03 | + | + | + | + |
| 29 | 7531.6 | 26.66 | + | + | + | + |
| 30 | 0 | Undetectable | Negative | Negative | Negative | Negative |
| 31 | 80.3 | 35.05 | + | + | + | + |
| 32 | 44742.4 | 20.78 | + | + | + | + |
| 33 | 1383.6 | 31.075 | + | + | + | + |
| 34 | 46.4 | Undetectable | + | + | + | + |
| 35 | 10717.3 | 26.995 | + | + | + | + |
| 36 | 1064.8 | 27.075 | + | + | + | + |
| 37 | 6112.8 | 28.145 | + | + | + | + |
| 38 | 571.8 | 28.18 | + | + | + | + |
| 39 | 26.7 | Undetectable | + | + | Negative | Negative |
| 40 | 1585.5 | 31.49 | + | + | + | + |
| 41 | 0 | Undetectable | Negative | Negative | Negative | Negative |
| 42 | 0 | Undetectable | Negative | Negative | Negative | Negative |
| 43 | 0 | Undetectable | Negative | Negative | Negative | Negative |
| 44 | 0 | Undetectable | Negative | Negative | Negative | Negative |
| 45 | 0 | Undetectable | Negative | Negative | Negative | Negative |
| 46 | 0 | Undetectable | Negative | Negative | Negative | Negative |
| 47 | 0 | Undetectable | Negative | Negative | Negative | Negative |
| 48 | 0 | Undetectable | Negative | Negative | Negative | Negative |
| 49 | 0 | Undetectable | Negative | Negative | Negative | Negative |
| 50 | 0 | Undetectable | Negative | Negative | Negative | Negative |
| 51 | 0 | Undetectable | Negative | Negative | Negative | Negative |
| 52 | 0 | Undetectable | Negative | Negative | Negative | Negative |
| 53 | 0 | Undetectable | Negative | Negative | Negative | Negative |
| 54 | 0 | Undetectable | Negative | Negative | Negative | Negative |
| 55 | 0 | Undetectable | Negative | Negative | Negative | Negative |
| 56 | 0 | Undetectable | Negative | Negative | Negative | Negative |
| 57 | 0 | Undetectable | Negative | Negative | Negative | Negative |
| 58 | 0 | Undetectable | Negative | Negative | Negative | Negative |
| 59 | 0 | Undetectable | Negative | Negative | Negative | Negative |
| 60 | 0 | Undetectable | Negative | Negative | Negative | Negative |
| 61 | 0 | Undetectable | Negative | Negative | Negative | Negative |
| 62 | 0 | Undetectable | Negative | Negative | Negative | Negative |
| 63 | 0 | Undetectable | Negative | Negative | Negative | Negative |
| 64 | 0 | Undetectable | Negative | Negative | Negative | Negative |
| 65 | 0 | Undetectable | Negative | Negative | Negative | Negative |
| 66 | 0 | Undetectable | Negative | Negative | Negative | Negative |
| 67 | 0 | Undetectable | Negative | Negative | Negative | Negative |
| 68 | 0 | Undetectable | Negative | Negative | Negative | Negative |
| 69 | 0 | Undetectable | Negative | Negative | Negative | Negative |
| 70 | 0 | Undetectable | Negative | Negative | Negative | Negative |
| 71 | 0 | Undetectable | Negative | Negative | Negative | Negative |
| 72 | 0 | Undetectable | Negative | Negative | Negative | Negative |
| 73 | 0 | Undetectable | Negative | Negative | Negative | Negative |
| 74 | 0 | Undetectable | Negative | Negative | Negative | Negative |
| 75 | 0 | Undetectable | Negative | Negative | Negative | Negative |
| 76 | 0 | Undetectable | Negative | Negative | Negative | Negative |
| 77 | 0 | Undetectable | Negative | Negative | Negative | Negative |

[1] Fan Z, Xu L, Cao Y, et al. One-Pot Assay Based on CRISPR/Cas13a Technology for HEV RNA Point-of-Care Testing. J Med Virol. 2024;96(12):e70115. doi:10.1002/jmv.70115.

[2] Cao Y, Tian Y, Huang J, et al. CRISPR/Cas13-assisted carbapenem-resistant *Klebsiella pneumoniae* detection. J Microbiol Immunol Infect. 2024;57(1):118-127.


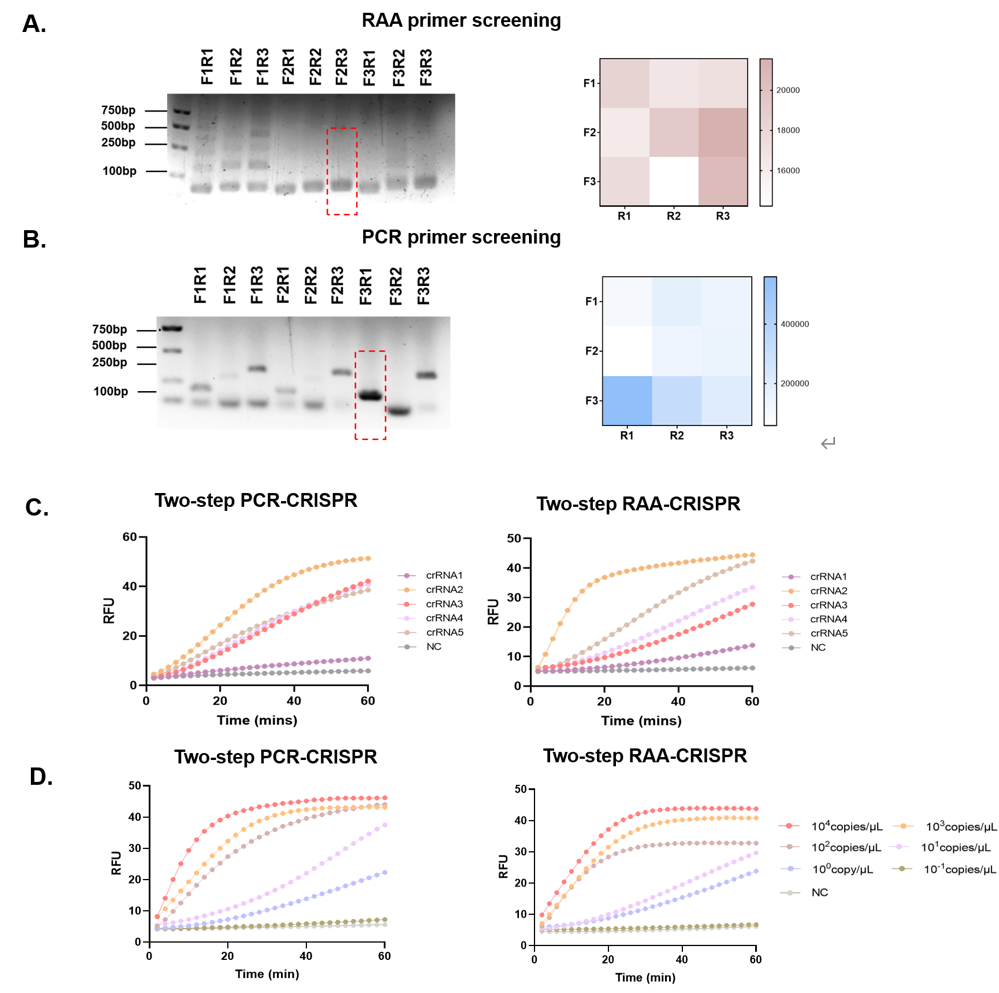


**Supplementary Figure 1 A.** RAA primer screening results: the left figure shows the agarose gel electrophoresis results, and the right figure shows the gray value analysis results. **B.** PCR primer screening results: the left figure shows the agarose gel electrophoresis results, and the right figure shows the gray value analysis results. **C.** Screening of crRNA. **D.** Sensitivity evaluation of the two-step PCR-CRISPR, two-step RAA-CRISPR assay using synthetic DNA.


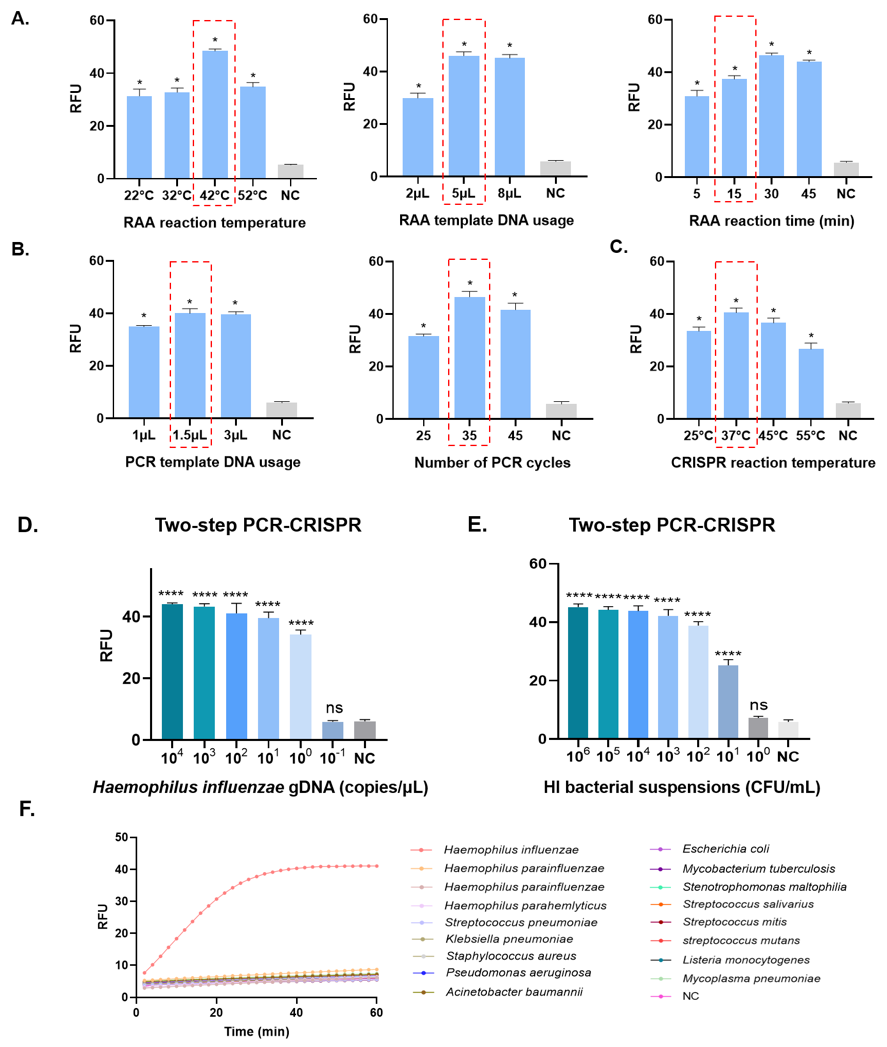


**Supplementary Figure 2 Optimization of the CRISPR/Cas13a detection system and evaluation of the two-step PCR-CRISPR assay for HI DNA.**

**A.** Optimization of the RAA reaction temperature (22°C, 32°C, 42°C, and 52°C), template volume (2, 5, and 8 μL) and reaction time (5, 15, 30, 45 min). **B.** Optimization of the PCR template volume (1, 1.5, and 3 μL) and number of PCR cycles (25, 35, and 45 cycles). **C.** Optimization of the CRISPR reaction temperature (25°C, 37°C, 45°C, and 55°C). **D, E.** Sensitivity evaluation of the two-step RAA-CRISPR assay using gradient dilutions of HI genomic DNA and HI bacterial suspensions. **F.** Specificity evaluation of the two-step RAA-CRISPR assay for detecting HI DNA using clinical bacterial strains. Data are representative of 3 independent experiments. (*P< 0.05, NC: negative control, NC is nuclease free water.)


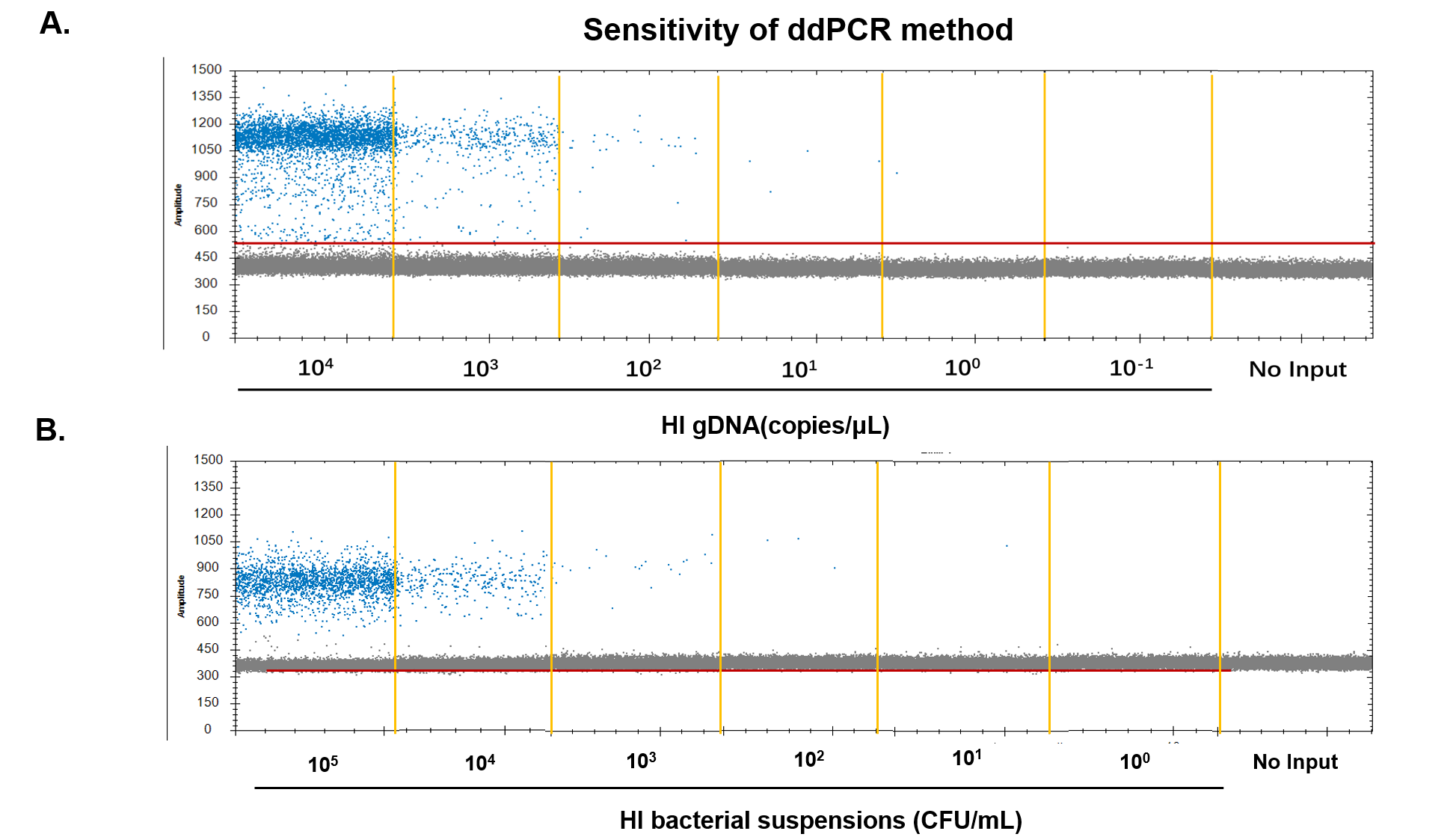


**Supplementary Figure 3 Sensitivity of the ddPCR assay to detect HI. A.** The 10-fold serial dilution of *Haemophilus influenzae* genomic DNA (gDNA) was detected by the ddPCR; the blue points represent the positive signal; the gray points represent the negative signal. **B.** The 10-fold serial dilution of HI bacterial suspensions was detected by the ddPCR; the blue points represent the positive signal; the gray points represent the negative signal.


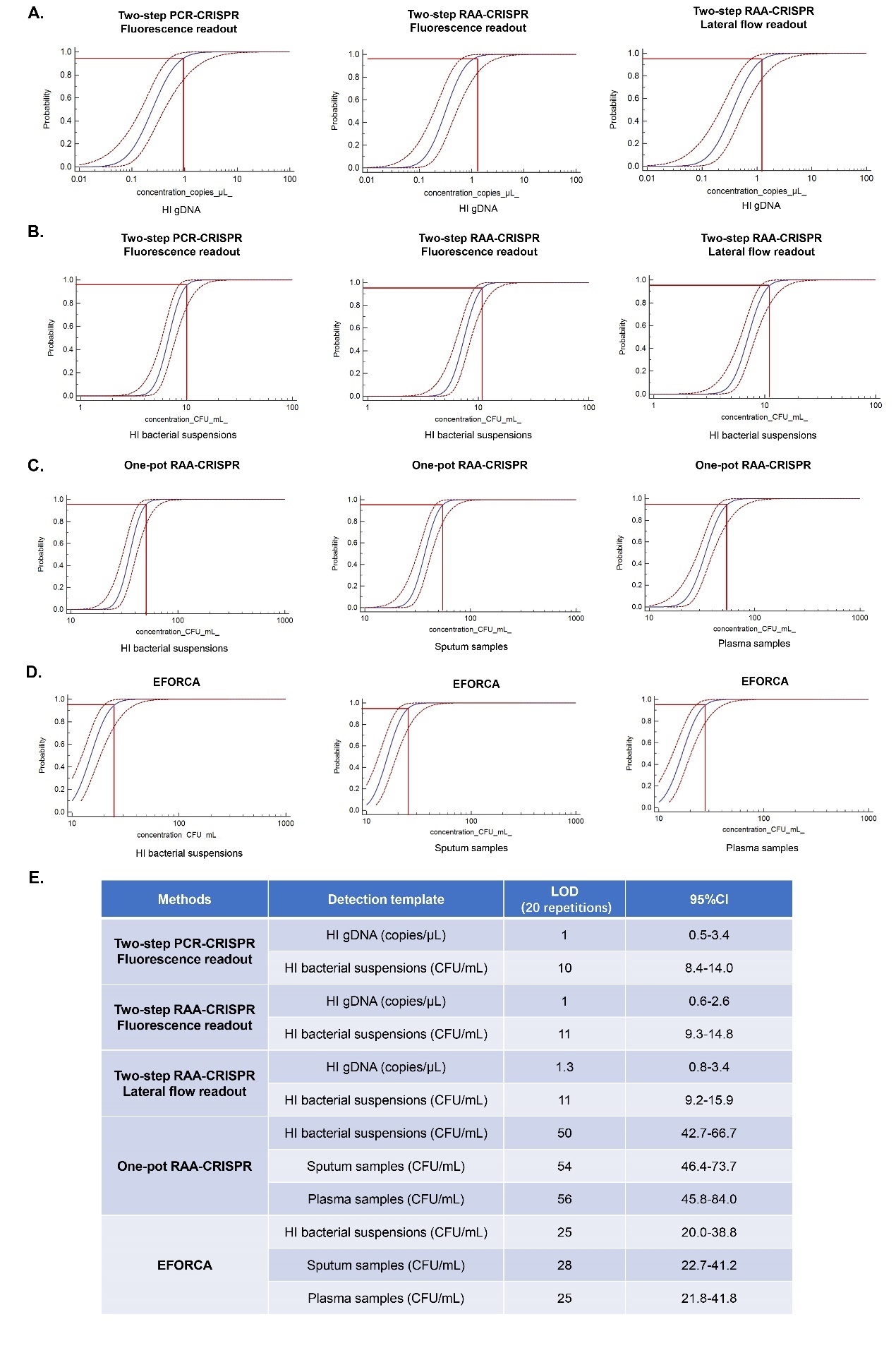


**Supplementary Figure 4. Determining the LoD of the two-step PCR-CRISPR assay, the two-step RAA-CRISPR assay, the one-pot RAA-CRISPR assay and the EFORCA using probit analysis of sigmoid curves.**

**A, B.** The LoDs for HI gDNA and HI bacterial suspensions in the two-step PCR-CRISPR assay, the two-step RAA-CRISPR assay (fluorescence readout) and the two-step RAA-CRISPR assay (lateral flow readout) were determined using probit analysis of sigmoid curves. Repeated these assays with different concentrations of HI gDNA (0.1, 1, 5, 10 copies/μL) were performed near the detection limits determined in the pre-experiment. The concentration of the bacterial suspension used were 1, 5, 10, 25, 50 CFU/mL. **C.** The probit analysis sigmoid curve was used to determine the LoD of the one-pot RAA-CRISPR assay for HI bacterial suspensions, simulated sputum samples and simulated plasma samples detection. The detected concentrations are 10, 25, 50 and 100 CFU/mL. **D.** The probit analysis sigmoid curve was used to determine the LoD of the EFORCA for HI bacterial suspensions, simulated sputum samples and simulated plasma samples detection. The detected concentrations are 10, 25, 50 and 100 CFU/mL. **E.** The summary of the LOD values of the CRISPR method established in this study. The X-axis represents the anticipated concentration, and the Y-axis shows the positivity rate across replicate tests. The blue line is the probit curve, and the red dashed line are 95% confidence interval (95% CI), the experiments were repeated 20 times for each concentration in the same parallel reactions.

**
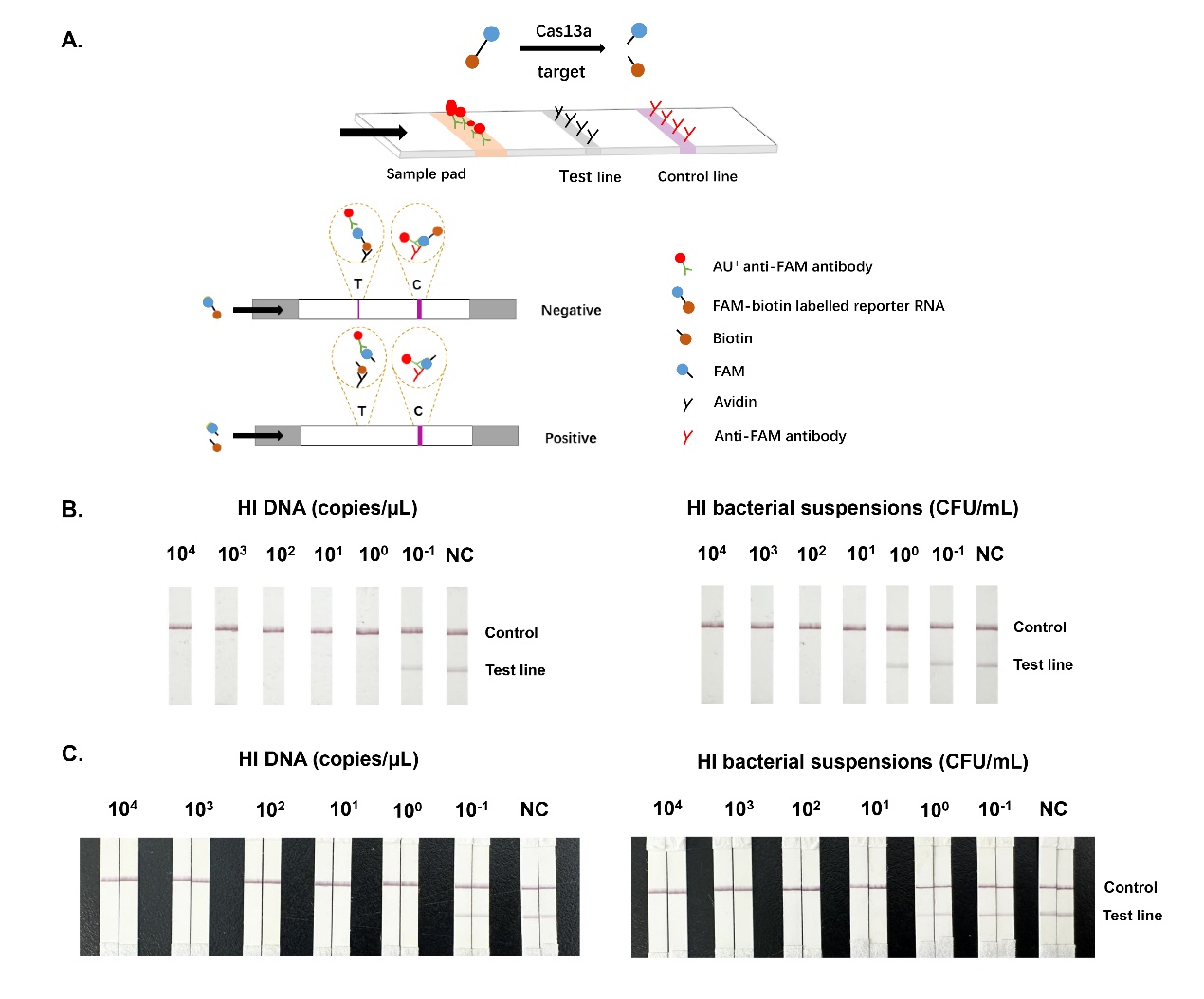
**

**Supplementary Figure 5 A.** The structure and schematic diagram of the lateral flow strip. **B.** Results of the RAA-CRISPR strip assay for serial dilutions of the synthetic DNA and bacterial suspensions (NC: non-infected samples). **C.** Results from the two remaining replicate experiments in Figure B.


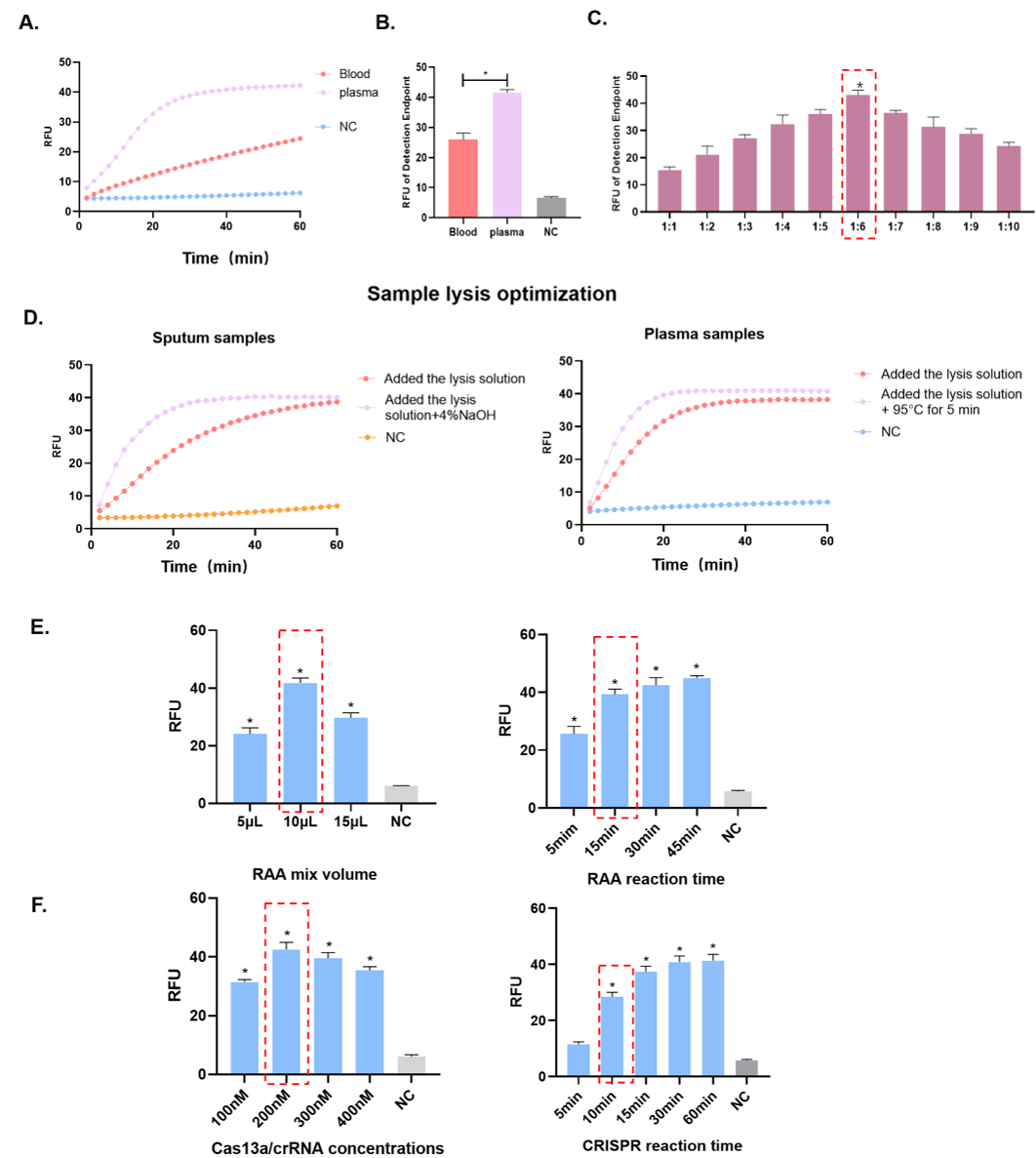


**Supplementary Figure 6 Optimization of the one-pot RAA-CRISPR assay. A, B.** Comparison of rapid lysis results of plasma and blood samples. The results of plasma and blood samples were compared by observing the fluorescent signal values detected by RAA-CRISPR. **C.** Lysis efficiency was quantified at different volume ratios of samples and lysis solutions. **D.** Lysis optimization of sputum samples and plasma samples. **E.** Optimization of the RAA mix volume (5, 10, and 15 μL), and reaction time (5, 15, 30 and 45 min). **F.** Optimization of the CRISPR reaction time (5, 10, 15, 30 and 60 min) and Cas13a/crRNA binary complex concentrations (100, 200, 300 and 400 mM). *p < 0.05.


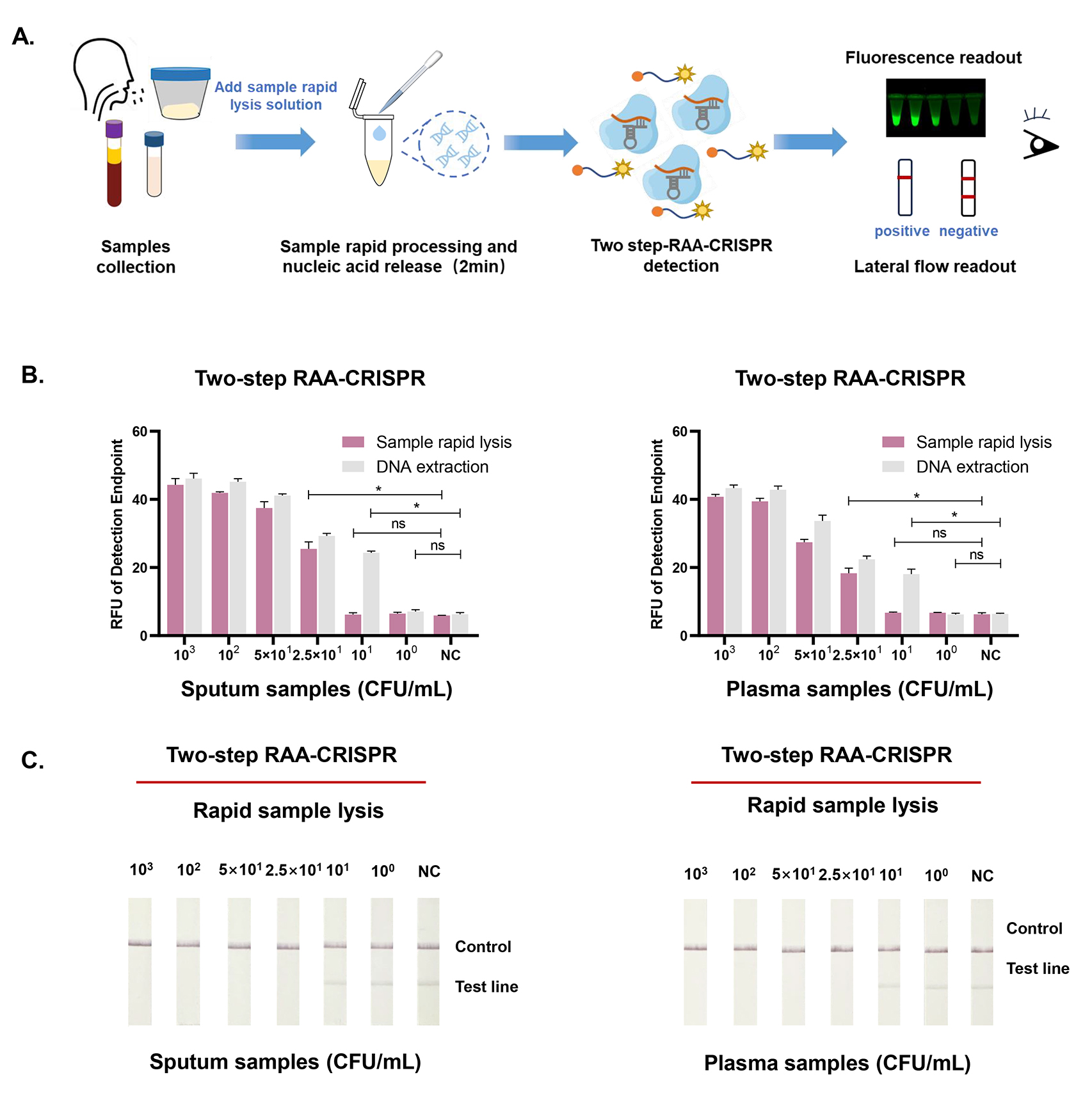


**Supplementary Figure 7** **Exploration and optimization of rapid sample lysis steps.**

**A.** Schematic diagram of the process of HI detection via rapid sample lysis. **B.** Results of the two step RAA-CRISPR assay (lateral flow readout) for rapidly processed samples from HI-simulated sputum samples and simulated plasma samples (NC: noninfected samples). **C.** Comparison of DNA extraction and rapid sample lysis in terms of sensitivity for simulated sputum samples and simulated plasma samples (NC: noninfected samples). The data are representative of 3 independent experiments. *p < 0.05.


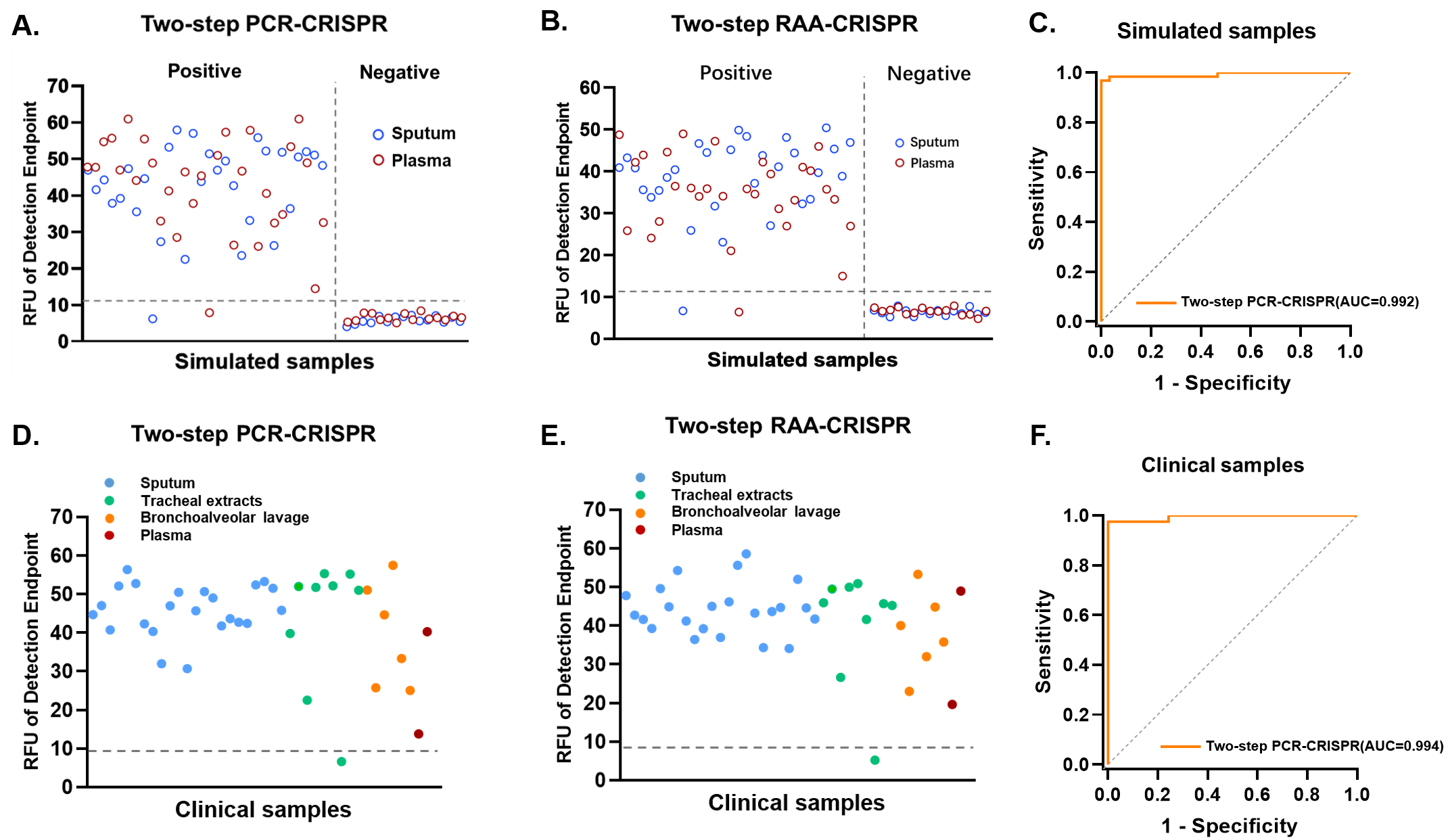


**Supplementary Figure 8. A, B.** The two-step PCR-CRISPR assay and two-step RAA-CRISPR assay result of HI in simulated samples. **C.** Analysis of the ROC curve from simulated samples testing, the orange lines represent the two-step PCR-CRISPR assay results. **D, E.** The two-step PCR-CRISPR assay and two-step RAA-CRISPR assay result of HI in clinical samples (40 positive samples). **F.** Analysis of the ROC curve from clinical samples testing, the orange lines represent the two-step PCR-CRISPR assay results. The data are representative of 3 independent experiments.


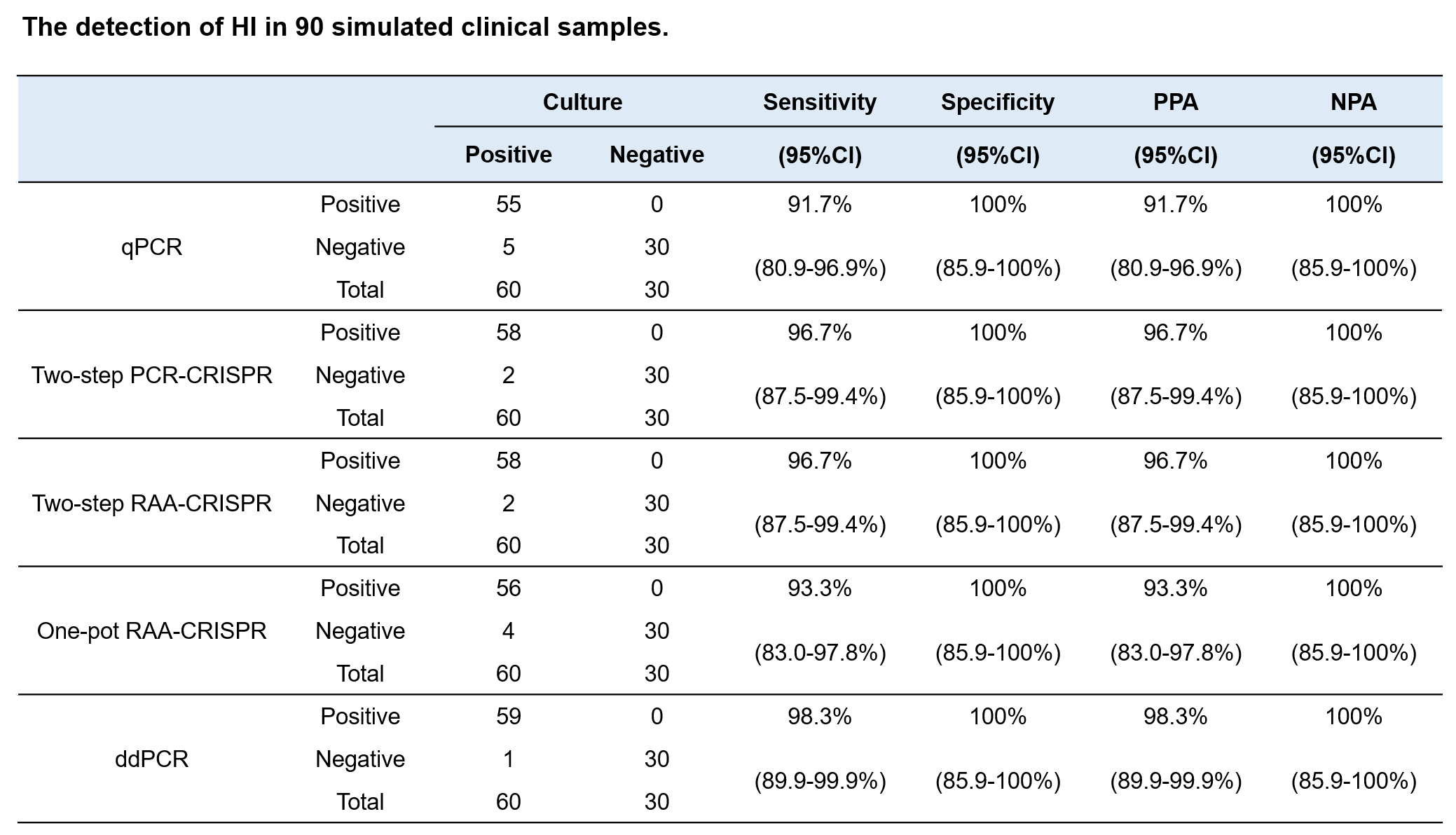


**Supplementary Figure 9** The detection of HI in 90 simulated clinical samples. Sensitivity, specificity, positive percent agreement (PPA), and negative percent agreement (NPA) were calculated using Clinical Calculator 1 (http://vassarstats.net/clin1.html#return).


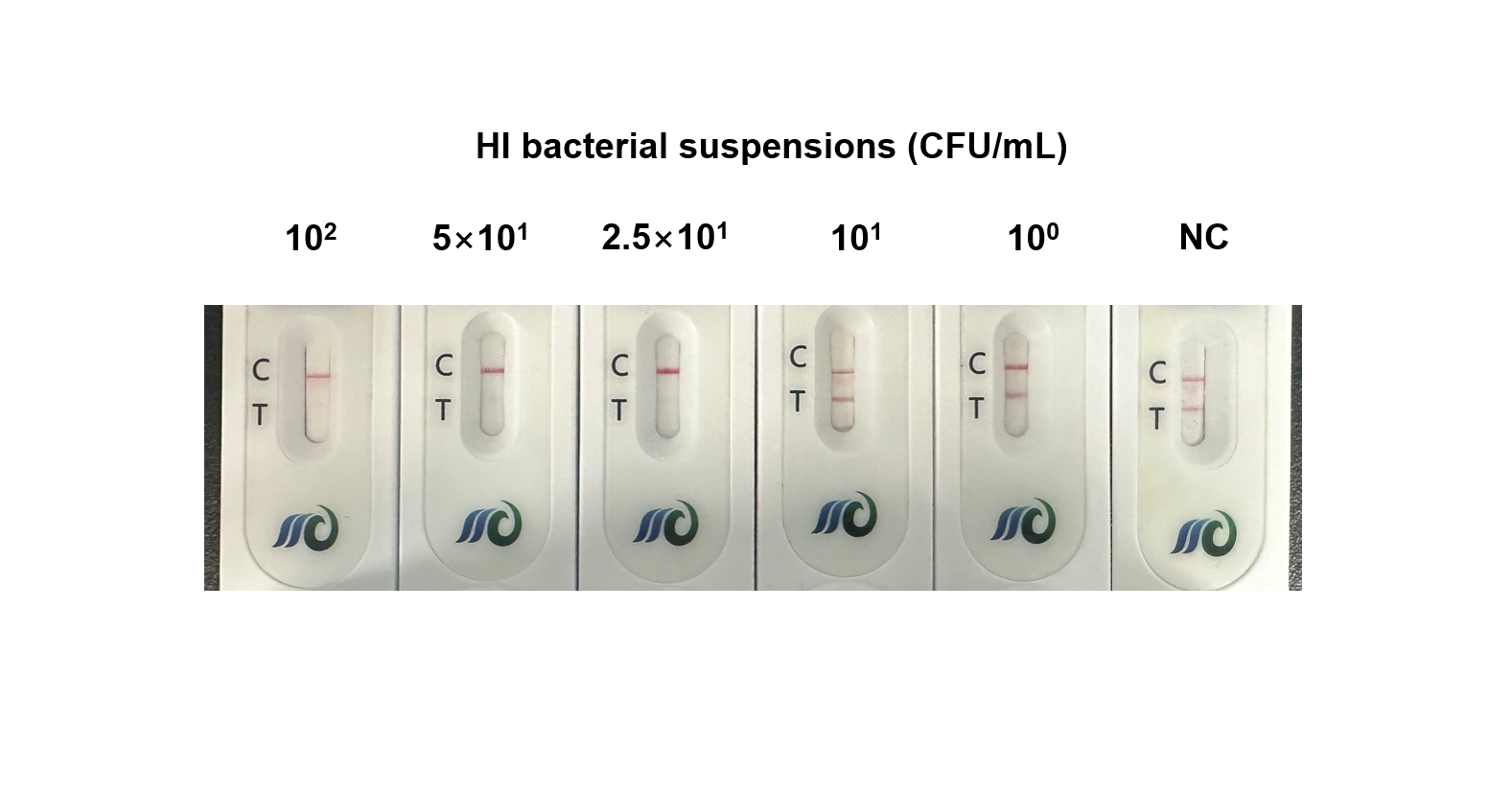


**Supplementary Figure 10** Results of the EFORCA for serial dilutions of HI bacterial suspensions (NC: nuclease-free sterile water).


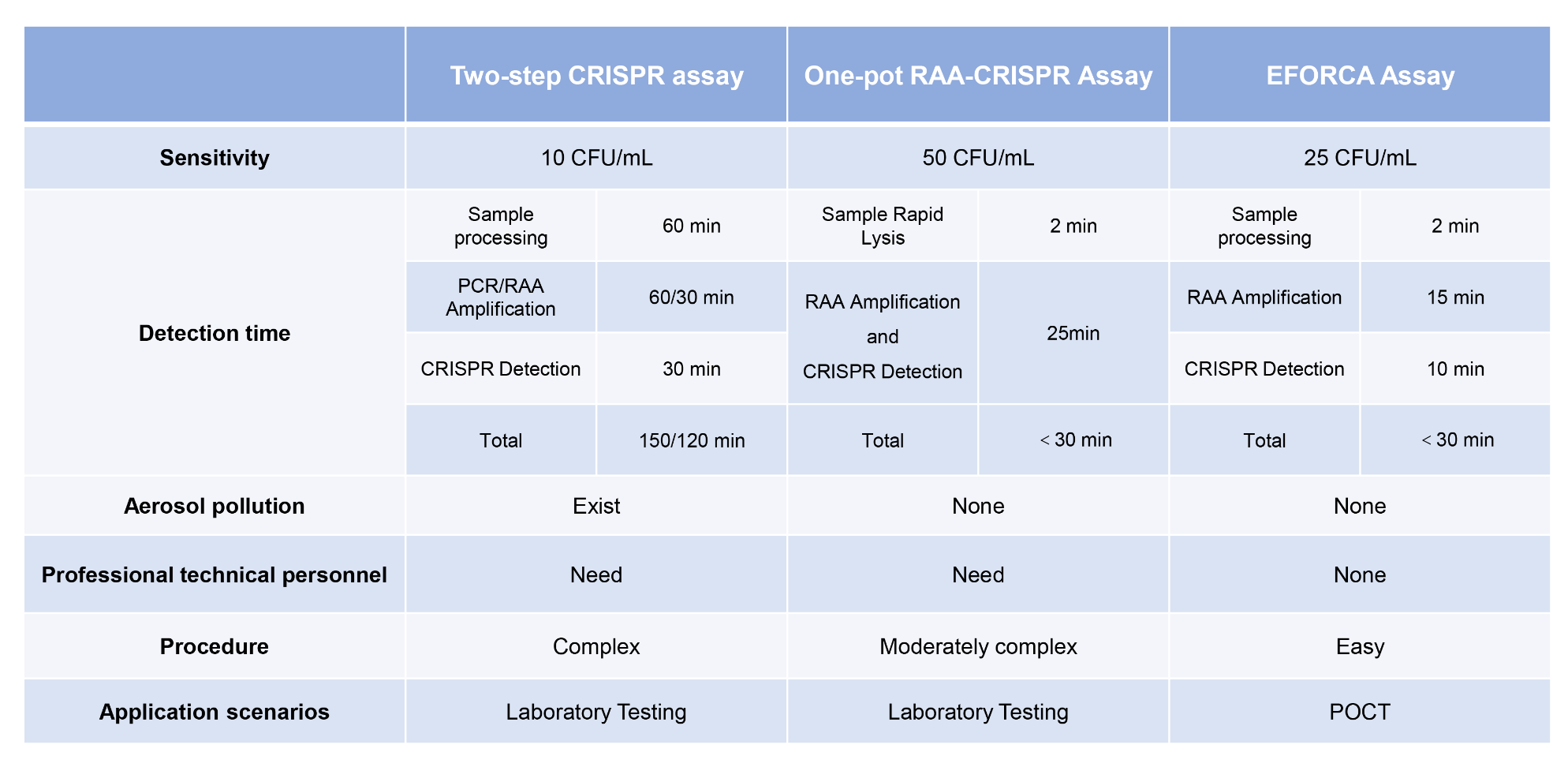


**Supplementary Figure 11.** Comparison between the two-step CRISPR assay, the one‐pot RAA-CRISPR assay and the EFORCA. The data are representative of 3 independent experiments.
